# A phase Ib trial of pembrolizumab plus paclitaxel or flat-dose capecitabine in 1st/2nd line metastatic triple-negative breast cancer

**DOI:** 10.1038/s41523-023-00541-2

**Published:** 2023-06-21

**Authors:** David B. Page, Joanna Pucilowska, Brie Chun, Isaac Kim, Katherine Sanchez, Nicole Moxon, Staci Mellinger, Yaping Wu, Yoshinobu Koguchi, Valerie Conrad, William L. Redmond, Maritza Martel, Zhaoyu Sun, Mary B. Campbell, Alison Conlin, Anupama Acheson, Reva Basho, Philomena McAndrew, Mary El-Masry, Dorothy Park, Laura Bennetts, Robert S. Seitz, Tyler J. Nielsen, Kimberly McGregor, Venkatesh Rajamanickam, Brady Bernard, Walter J. Urba, Heather L. McArthur

**Affiliations:** 1 Earle A. Chiles Research Institute, Providence Cancer Institute, Portland, OR USA; 2 Cedars Sinai Medical Center, Los Angeles, CA USA; 3 Ellison Institute for Transformative Medicine, Los Angeles, CA USA; 4 Oncocyte Corporation, Irving, CA USA; 5 UT Southwestern Medical Center, Dallas, TX USA

**Keywords:** Breast cancer, Tumour immunology, Predictive markers, Breast cancer, Breast cancer

## Abstract

Chemoimmunotherapy with anti-programmed cell death 1/ligand 1 and cytotoxic chemotherapy is a promising therapeutic modality for women with triple-negative breast cancer, but questions remain regarding optimal chemotherapy backbone and biomarkers for patient selection. We report final outcomes from a phase Ib trial evaluating pembrolizumab (200 mg IV every 3 weeks) with either weekly paclitaxel (80 mg/m^2^ weekly) or flat-dose capecitabine (2000 mg orally twice daily for 7 days of every 14-day cycle) in the 1st/2nd line setting. The primary endpoint is safety (receipt of 2 cycles without grade III/IV toxicities requiring discontinuation or ≥21-day delays). The secondary endpoint is efficacy (week 12 objective response). Exploratory aims are to characterize immunologic effects of treatment over time, and to evaluate novel biomarkers. The trial demonstrates that both regimens meet the pre-specified safety endpoint (paclitaxel: 87%; capecitabine: 100%). Objective response rate is 29% for pembrolizumab/paclitaxel (*n* = 4/13, 95% CI: 10–61%) and 43% for pembrolizumab/capecitabine (*n* = 6/14, 95% CI: 18–71%). Partial responses are observed in two subjects with chemo-refractory metaplastic carcinoma (both in capecitabine arm). Both regimens are associated with significant peripheral leukocyte contraction over time. Response is associated with clinical PD-L1 score, non-receipt of prior chemotherapy, and the H&E stromal tumor-infiltrating lymphocyte score, but also by a novel 27 gene IO score and spatial biomarkers (lymphocyte spatial skewness). In conclusion, pembrolizumab with paclitaxel or capecitabine is safe and clinically active. Both regimens are lymphodepleting, highlighting the competing immunostimulatory versus lymphotoxic effects of cytotoxic chemotherapy. Further exploration of the IO score and spatial TIL biomarkers is warranted. The clinical trial registration is NCT02734290.

## Introduction

Chemotherapy plus anti-programmed death 1/ligand 1 (anti-PD-1/L1) has become a standard-of-care treatment for selected patients with triple-negative breast cancer (TNBC). For example, in the Keynote-355 phase III trial, the addition of anti-PD-1 (pembrolizumab) to chemotherapy (paclitaxel, nab-paclitaxel, or gemcitabine/carboplatin) improved progression-free survival (PFS: 9.7 mo versus 5.6 mo, hazard ratio [HR] = 0.72, 95% confidence interval [CI]: 0.50–0.88)^1^ and overall survival (OS: 23.0 mo v. 16.1 mo, HR = 0.73, 95% CI: 0.55–0.95)^1^ in women with previously untreated metastatic PD-L1-positive TNBC. In a similar front-line phase III trial (IMpassion130), the addition of anti-programmed death ligand 1 (atezolizumab) to nab-paclitaxel improved PFS (7.5mo v. 5.3mo, HR 0.80, 95% CI: 0.69–0.92) in patients with PD-L1-positive disease, and improved OS in an informal analysis^2–4^. In stage II/III TNBC, pembrolizumab was shown in the phase II I-SPY2 and phase III Keynote-522 trials to improve pathological complete response (pCR) rate and event-free survival when combined with curative-intent neoadjuvant anthracycline/taxane-based chemotherapy^5–7^. Similarly, in the phase III IMpassion031 trial, the addition of PD-L1 blockade with atezolizumab to neoadjuvant anthracycline/taxane-based chemotherapy improved pCR rates in stage II/III TNBC^8^.

These data have prompted the United States Food and Drug Administration (FDA) to approve chemotherapy plus pembrolizumab for patients with stage II/III TNBC and PD-L1-positive stage IV TNBC. However, several other clinical trials have failed to confirm the efficacy of chemotherapy with immunotherapy. For example, the phase III IMpassion131 study, despite a very similar study design to IMpassion130, failed to demonstrate the efficacy of atezolizumab when combined with front-line weekly paclitaxel^9^. Trials evaluating other chemoimmunotherapy combinations in later lines have failed to demonstrate superiority to chemotherapy alone; in particular, a phase II trial of pembrolizumab plus capecitabine failed to demonstrate an improvement in efficacy when compared to historical controls who received capecitabine monotherapy^10^.

Here, we report the results of a phase Ib investigator-initiated trial evaluating pembrolizumab combined with one of two standard-of-care palliative chemotherapy regimens, weekly paclitaxel or oral capecitabine (7d on/7d off)11 per treating physician’s choice, for 1st or 2nd line palliation of metastatic TNBC. The primary objective was to demonstrate clinical feasibility of these chemoimmunotherapy regimens; however, we also aimed to conduct comprehensive immunological monitoring to identify novel predictive biomarkers, and to compare immunological effects attributed to the two chemoimmunotherapy regimens. We present the final clinical safety and efficacy outcomes, including the treatment of four women with metaplastic breast cancer, a rare but aggressive form of metastatic TNBC. We then summarize exploratory associations of immune-based biomarkers with response, including a gene expression signature validated in early-stage TNBC that has not yet been tested in mTNBC (the 27-gene IO score), and a novel approach for characterizing tumor-infiltrating lymphocytes (TILs) according to both density and spatial distribution. Finally, we illustrate the impact of these chemoimmunotherapy regimens on peripheral immune cell quantity and diversity over time, providing mechanistic insight into the discordant clinical outcomes observed across chemoimmunotherapy trials in metastatic TNBC.

## Results

### Patients

**Fig. 1:**
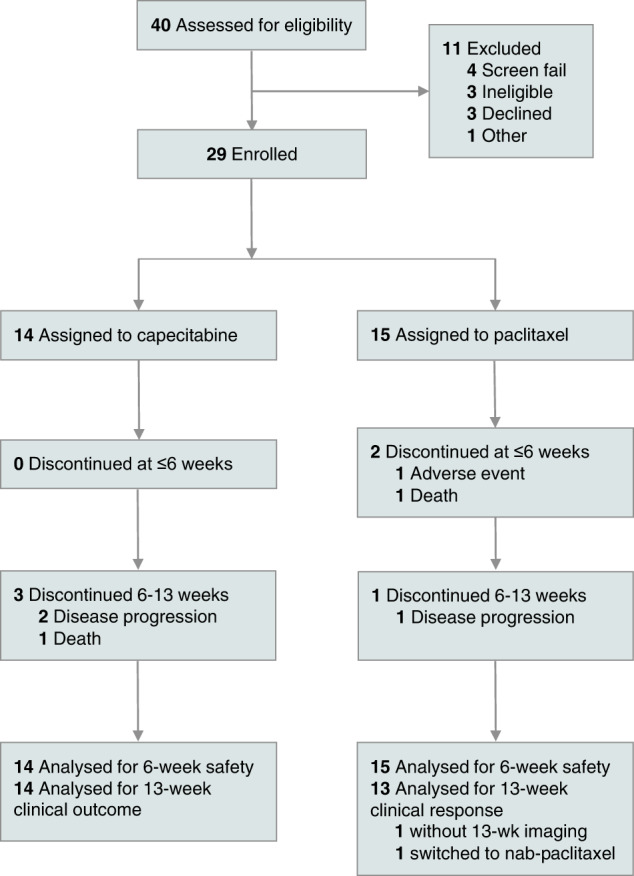
CONSORT diagram illustrating enrollment of subjects.

Between 2016 and 2018, 28 women and 1 man with mTNBC were enrolled. The average age was 59 years (39–85) (refer to Fig. 1 for CONSORT diagram). Demographic information is summarized in Table 1. Of the 15 patients treated with paclitaxel, two were not evaluable for efficacy (the first because week 12 imaging assessment was missed, and the second because the patient switched from paclitaxel to nab-paclitaxel). All 14 patients treated with capecitabine were evaluable for efficacy and safety. 59% of subjects (*n* = 17/29) received treatment in the first-line setting and 41% of subjects (*n* = 12) received treatment in the second-line setting. Among the 7 (*n* = 7/29, 24%) subjects who received no prior chemotherapy in the neo/adjuvant or metastatic setting, 5 had de novo metastatic disease (Table 1).

**Table 1:** Demographic data.

Clinical value	Capecitabine (*n* = 14)	Paclitaxel (*n* = 15)
Age, mean (SD), y	63 (8.7)	56 (12.5)
RECIST 1.1 tumor burden, cm		
Median (range)	7.0 (1.1–12.4)	4.8 (1.5–17.5)
Mean (SD)	6.5 (3.4)	8.0 (5.6)
DDFI, median (range)	17.8 (0.6–71.3)	46.5 (10.7–96.6)
Ethnicity		
Caucasian	13 (93%)	9 (60%)
Non-Caucasian	1 (7%)	6 (40%)
ECOG score, no. (%)		
0	5 (36%)	4 (27%)
1	9 (64%)	11 (73%)
Liver metastases	2 (14%)	4 (27%)
De novo metastatic disease	1 (7%)	4 (27%)
Line of treatment		
First line	8 (43%)	9 (60%)
Second line^a^	6 (57%)	6 (40%)
Prior (neo)-adjuvant chemo		
None	1 (7%)	6 (40%)
Anthracycline-based	10 (71%)	6 (40%)
TC	3 (21%)	3 (20%)

*RECIST 1.1* Response Evaluation Criteria in Solid Tumors guideline, version 1.1, *DDFI* distant disease-free interval, *ECOG* Eastern Cooperative Oncology Group, *mTNBC* metastatic triple-negative breast cancer, *ACT* doxorubicin, cyclophosphamide, and paclitaxel, *TC* docetaxel, cyclophosphamide.

^a^(neo)-adjuvant systemic therapy counted as one line if received <6 months from study enrollment.

### Safety

The primary endpoint of acceptable safety was achieved in both groups, with 87% (*n* = 13/15) of patients completing at least two cycles of treatment of paclitaxel/pembrolizumab therapy without discontinuation of therapy, and 100% (*n* = 14/14) of patients completing at least two cycles of capecitabine/pembrolizumab therapy without discontinuation. One patient discontinued therapy prior to completion of cycle 2 in the paclitaxel group, due to a hypersensitivity reaction attributed to paclitaxel infusion. There was one death in the paclitaxel group due to sepsis prior to completion of two cycles of therapy.

In the paclitaxel/pembrolizumab arm, all patients received dexamethasone 10–12 mg IV on cycle 1 day 1. Typically, this was continued until cycle 1 d 15, when a taper to half the previous dose was attempted with discontinuation thereafter at the treating clinician’s discretion. Accordingly, 43% (*n* = 6/14) of evaluable patients continued dexamethasone beyond cycle 1. Exploratory assessment of peripheral blood immunologic changes is presented in Supplementary Fig. 3; however, there were no obvious trends related to dexamethasone discontinuation.

Table 2 illustrates the incidence and attribution of adverse events, and Supplementary Table 1 summarizes all events attributed to pembrolizumab. Grade 3–4 adverse attributed to pembrolizumab included adrenal insufficiency (*n* = 1/29), anemia (*n* = 1/29), fatigue (*n* = 2/29), hyperbilirubinemia (*n* = 1/29), hyperglycemia (*n* = 1/29), motor neuropathy (*n* = 1/29), and sepsis (*n* = 1/29). Chemotherapy dose reductions were common in both arms, and effective in mitigating chemotherapy-attributed toxicities (Supplementary Table 2). Most patients in the capecitabine/pembrolizumab arm required dose reduction by week 12, with the most common dose being 1500 mg PO BID. In the capecitabine arm, 43% of patients experienced grade 1–2 diarrhea and 14% experienced grade 3–4 diarrhea. All cases of diarrhea were self-limited or responded to supportive medications such as loperamide. None of these cases required diagnostic procedures or systemic corticosteroids. Supplementary Table 3 provides guidance on the management of diarrhea/colitis, derived from the protocol.

**Table 2:** Physician-reported maximum adverse events experienced by ≥20% of subjects.

A: Capecitabine arm (*n* = 14)				
Event Term	All Grades % (*n*)	Grade 3–4 % (*n*)	Attributed to pembro	Attributed to capecitabine
Hand/Foot syndrome	71% (10)			64% (9)
Fatigue	64% (9)	7% (1)		57% (8)
Dyspnea	64% (9)	7% (1)		
Cough	57% (8)			
Diarrhea	57% (8)	14% (2)		57% (8)
Headache	50% (7)			
Nausea/vomiting	50% (7)	7% (1)		36% (5)
Anorexia	50% (7)	7% (1)		14% (2)
Hyponatremia	50% (7)	21% (3)		7% (1)
Anemia	43% (6)	14% (2)		21% (3)
Dry skin	36% (5)			14% (2)
Hypokalemia	36% (5)			14% (2)
Low WBC count	36% (5)	28% (4)		21% (3)
Wheezing	29% (4)			
Constipation	29% (4)		7% (1)	14% (2)
Dry mouth	29% (4)		7% (1)	7% (1)
Fever	29% (4)			
Pleural effusion	29% (4)			
Abdominal pain	29% (4)	7% (1)		21% (3)
Mucositis	21% (3)			14% (2)
Heartburn	21% (3)			14% (2)
Facial/body edema	21% (3)			7% (1)
Hypothyroidism	21% (3)		21% (3)	
Hypomagnesemia	21% (3)			7% (1)

B: Paclitaxel arm (*n* = 15)				
Event Term	All Grades % (*n*)	Grade 3–4 % (*n*)	Attributed to pembro	Attributed to paclitaxel
Nausea/vomiting	67% (9)	13% (20)		40% (6)
Diarrhea	53% (8)			20% (3)
Neuropathy	53% (8)	7% (1)	7% (1)	40% (6)
Fatigue	53% (8)	13% (2)		46% (7)
Anemia	40% (6)	27% (4)		27% (4)
Dyspnea	40% (6)	20% (3)		
Facial/body edema	40% (6)	7% (1)		7% (1)
Mucositis	33% (5)			7% (1)
Other pain	33% (5)			7% (1)
Alopecia	33% (5)	7% (1)		33% (5)
Other rash	33% (5)	7% (1)		14% (2)
Fever	33% (5)	7% (1)		
Anorexia	27% (4)			20% (3)
Maculopapular rash	27% (4)			20% (3)
Abdominal pain	27% (4)	7% (1)		
Hypokalemia	27% (4)	7% (1)		7% (1)
Dysgeusia	20% (3)			20% (3)
Hypomagnesemia	20% (3)			
Pleural effusion	20% (3)	7% (1)		
Hyponatremia	20% (3)	14% (2)		

Toxicities are as defined by the CTCAEv4.0. Adverse events were documented at all scheduled assessments weekly for the first 12 weeks, every 3 weeks thereafter and more frequently as clinically indicated.

*CTCAEv4.0* Common Terminology Criteria for Adverse Events version 4.0, *Other pain* non-attributable to cancer, *Other rash* non consistent with hand/foot syndrome, maculopapular rash.

### Efficacy

Among 13 patients evaluable for efficacy in the pembrolizumab/paclitaxel arm, there was a 12-week ORR of 31% (95% CI 10–61%), a 12-week clinical benefit rate (CBR, CR + PR + SD) of 38% (95% CI: 15–67%; *n* = 2/13 CR, *n* = 2/13 PR, *n* = 1/13 SD) and a median PFS of 83 days. At week 24, the ORR was 15% (95% CI: 0–36%) and CBR was 15% (95% CI: 0–36%, *n* = 1/13 CR, *n* = 1/13 PR,). Two of the 15 patients treated on this arm were not evaluable for efficacy, but both experienced objective responses. One was replaced because week 12 imaging was not available; however, she eventually experienced a partial response (PFS 308 days). A second patient required a switch to nab-paclitaxel because of infusion reaction and therefore was not evaluable for the week 12 objective response endpoint; they went on to achieve a CR, with ongoing response maintained on pembrolizumab monotherapy (PFS 1093+ days).

**Fig. 2:**
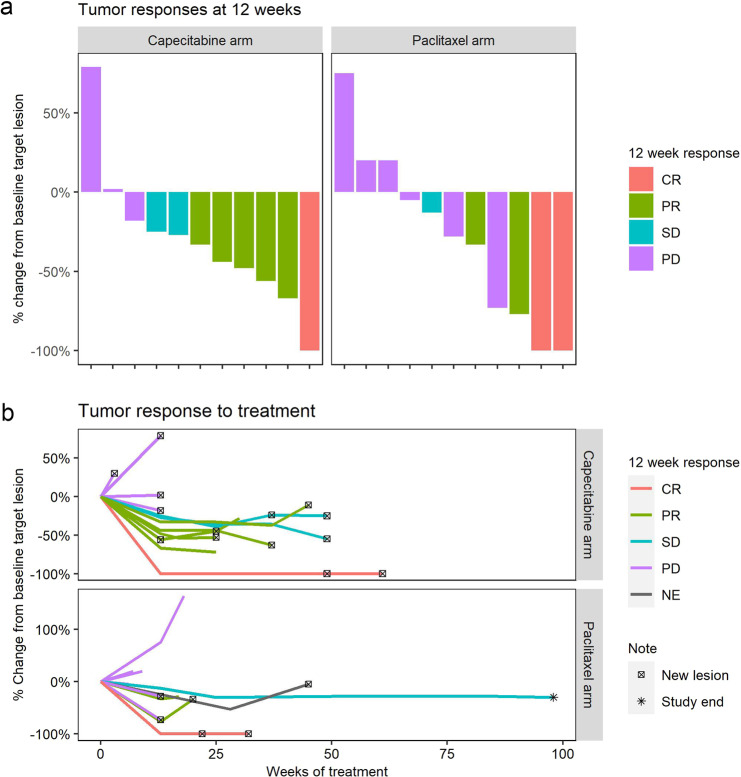
Tumor response. **a** Waterfall plot of percent change in tumor size at 12 weeks by RECIST 1.1 criteria in capecitabine and paclitaxel arms, respectively. Two patients are not depicted in capecitabine arm and 3 in paclitaxel arm due to progression of disease prior to week 12. Some subjects experienced RECIST1.1 progression due to progression of non-target or new lesions, despite radiographic shrinkage of target lesions. **b** Spider plot of percent change in tumor size, by RECIST 1.1 criteria in capecitabine and paclitaxel arms, respectively. RECIST 1.1 Response Evaluation Criteria in Solid Tumors guidelines, version 1.1, CR complete response, PR partial response, SD stable disease, PD progression of disease, NE not evaluable.

**Fig. 3:**
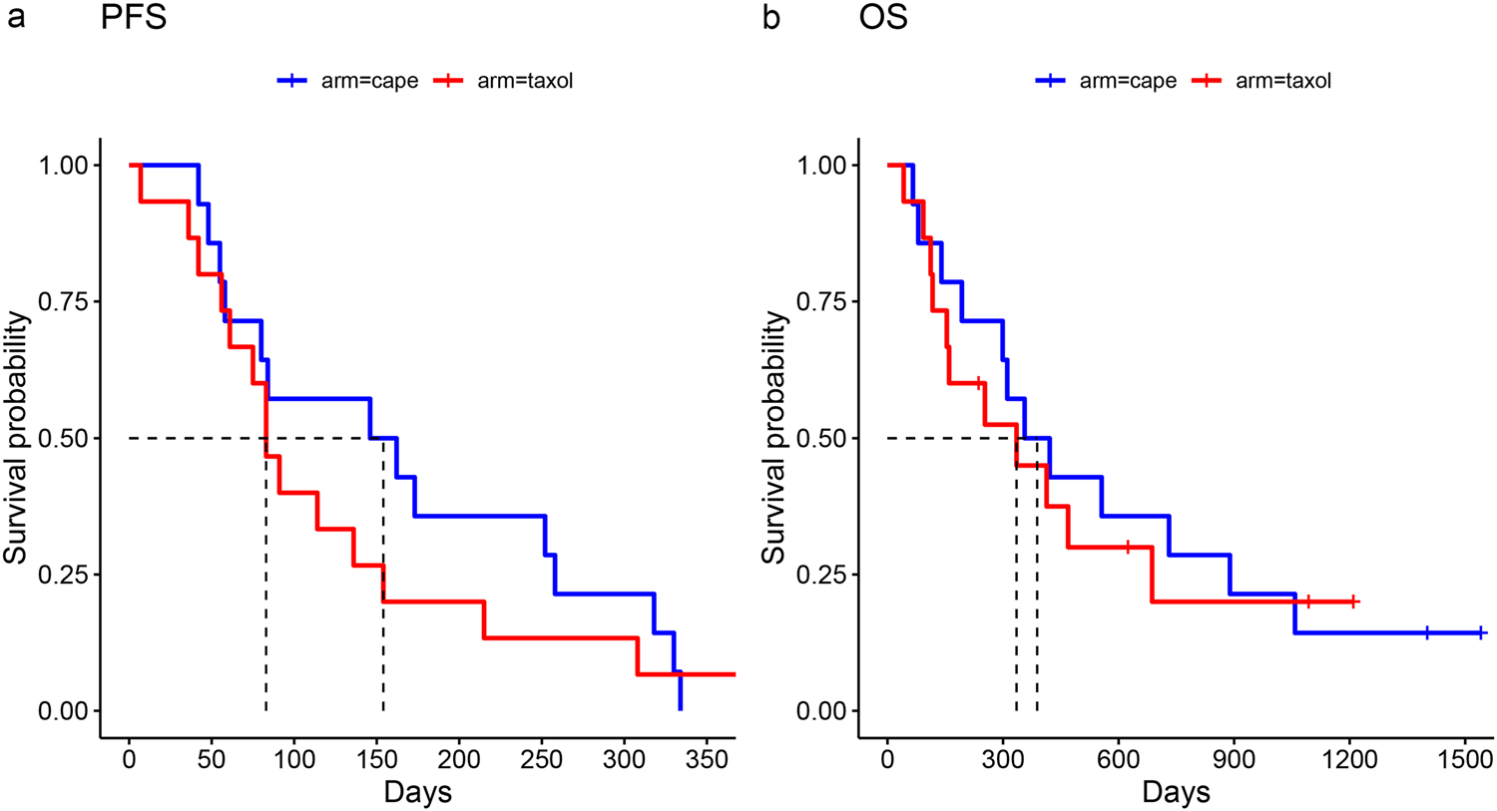
Kaplan–Meier survival curves. Kaplan–Meier estimates of progression-free survival (**a**) and overall survival (**b**). Dotted lines represent the median survival. Statistical comparisons of the survival curves are not performed because the study is not randomized or controlled for covariates. Cape Capecitabine, PFS progression-free survival, OS overall survival.

Among 14 evaluable patients in the pembrolizumab/capecitabine arm, there was a 12-week ORR of 43% (95% CI: 18–71%), a 12-week CBR of 57% (95% CI: 29–82%, *n* = 1/14 CR, *n* = 5/14 PR, *n* = 2/14 SD), and a median PFS of 155 days. At week 24, the ORR was 29% (95% CI: 8–58%) and CBR was 43% (*n* = 1/14 CR, *n* = 3/14 PR, *n* = 2/14 SD, 95% CI: 18–71%). The waterfall and spider plots of responses for each arm, and the Kaplan-Meier PFS and OS curves, are illustrated in Figs. 2, 3. Efficacy of the two arms cannot be directly compared because the trial was not randomized, and the arms were imbalanced for potential confounding factors including race (non-Caucasian: 40% paclitaxel/pembro v. 7% cape/pembro), liver metastases (27% v. 14%), de novo disease (27% v. 7%), treatment line (first-line: 60% v. 43%), and distant disease-free interval (i.e., time from surgery to stage IV recurrence; paclitaxel/pembro: median 47 m, capecitabine/pembro: median 18 m, log-rank *p* = 0.08, Table 1). However, the PFS/OS curves of the two arms appear comparable, with a possibility of improved PFS and OS in the pembrolizumab/capecitabine arm.

**Fig. 4:**
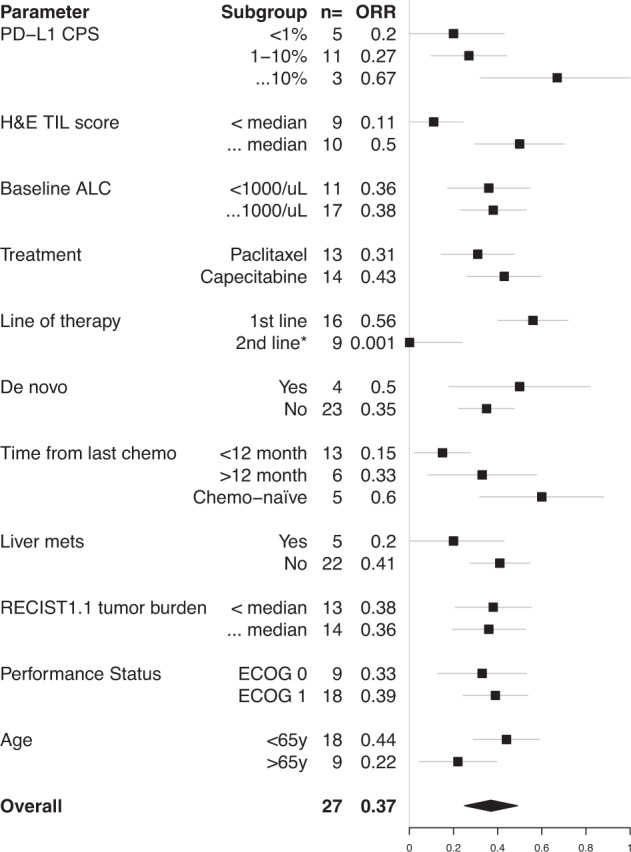
Associations of response with clinical parameters. Intervals represent the 80% confidence interval of the point estimate of response rate. PD-L1: programmed death ligand 1; CPS combined positive score, TIL tumor-infiltrating lymphocytes, ALC absolute lymphocyte count, RECIST response evaluation criteria in solid tumors, *(neo)-adjuvant systemic therapy counted as one line if received <6 months from study enrollment.

Figure 4 illustrates clinical outcomes stratified by patient or tumor-level covariates. Limited sample size requires that these findings be presented for exploratory purposes without statistical testing or multivariate analysis. Consistent with previous studies, responses were frequent among patients with PD-L1-high tumors, without liver involvement, or whose tumors had elevated stromal tumor-infiltrating lymphocytes (sTILs)^11,12^. Another factor posited to influence chemoimmunotherapy efficacy is prior exposure to systemic chemotherapy. In this clinical trial, no objective responses or clinical benefits were observed among subjects treated in the second-line setting, whereas responses were observed in the first-line setting. Responses were greatest among chemotherapy-naïve patients (i.e., subjects with no preceding curative-intent or palliative chemotherapy, ORR 60% [*n* = 3/5, 95% CI 15–95%]), whereas responses were lower among subjects with distant chemotherapy exposure in the curative-intent setting (chemotherapy at least 12 months prior to enrollment, ORR 33% [*n* = 2/6, 95% CI 4–78%]), and responses were lowest among patients receiving recent chemotherapy (<12 months, ORR 15% [*n* = 2/13, 95% CI 2–45%]). Previous exposure to the same class of chemotherapy in the curative setting appeared to influence response, as evidenced by lower responses observed amongst taxane-exposed patients in the pembrolizumab/paclitaxel arm (taxane-exposed: 22% ORR [*n* = 2/9, 95% CI 3–60%], 33% CBR; taxane-naïve: 60% ORR [*n* = 3/5, 95% CI 15–95%], 60% CBR). No patients in this group were exposed to curative-intent capecitabine as it was not yet standard-of-care per the CREATE-X trial^13^.

### Activity in metaplastic TNBC

**Fig. 5:**
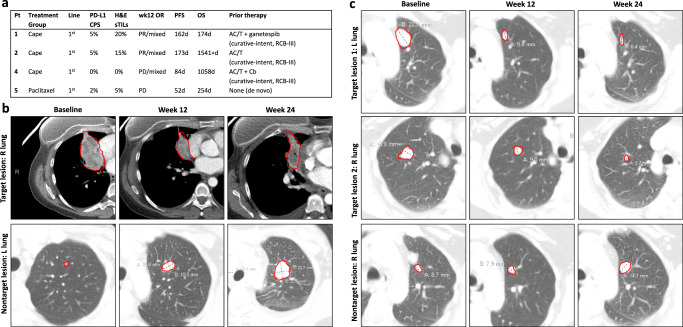
Outcomes in metaplastic TNBC patients. **a** Summary of clinical findings of metaplastic patients; **b** radiographic response of patient 1. Findings overall demonstrate a partial response, but with an initial non-target left lung nodule gradually increasing in size; **c** radiographic response of patient 2, showing a mixed, but overall partial response. Cape capecitabine, CPS combined positive score, H&E hematoxylin & eosin, sTILs stromal tumor-infiltrating lymphocytes, PR partial response, PD progression of disease, R right, L left, AC/T doxorubicin, cyclophosphamide, and paclitaxel, Cb carboplatin, RCB residual cancer burden score.

Four patients with metaplastic TNBC were enrolled, three in the capecitabine arm, and one in the paclitaxel arm. Responses are summarized in Fig. 5. We observed two clinical responses (PR at 12 weeks), both in the capecitabine arm (Fig. 5). Response durations were 162 and 173 days. Images of both patients showed evidence of mixed response, with some lesions shrinking and others growing (Fig. 5B). Both patients had tumors with borderline PD-L1 CPS scores (case 1: CPS = 5, case 2: CPS = 5) which would be considered PD-L1-negative using the paradigm established by the Keynote-355 trial^14^. One of the 2 non-responding tumors (patient 3, capecitabine arm) exhibited mixed tumor growth and regression, but overall PD by RECIST1.1 criteria. Additional details regarding these cases are summarized in a recently published case series^15^.

### Exploratory immune-based biomarker assessment

#### Whole blood immune cell assessment

Flow cytometry and TCR sequencing were used to explore univariate associations of baseline peripheral blood immune profile with week 12 OR, the secondary outcome of the study. Clinical responses according to these biomarkers are summarized in Supplementary Figs. 4, 5, with ORRs stratified according to above/below the median for the biomarker. There was a trend toward higher B cell and CD4 + T cell counts among patients whose tumors had an objective response^16–18^.

TCR diversity is influenced significantly by chemotherapy^19^, is prognostic of survival in metastatic breast cancer^20^, and may predict response to anti-PD-1/L1 in other cancer types^21–23^. There was a trend toward greater baseline peripheral TCR richness, a surrogate metric of T-cell clonal diversity, in patients who experienced an objective response (Supplementary Fig. 6).

#### Tumor genomics

The diversity of tumor-infiltrating T cells, and bulk tumor gene expression profiling, were evaluated by TCR sequencing and RNA exome sequencing, respectively. Results are illustrated in Supplementary Figs. 7–13. We observed no strong associations of previously described T cell diversity metrics (i.e., clonality, richness, TCR templates) or previously described RNA cell type signatures with response (Supplementary Fig. 7). We identified individual genes that were differentially expressed among responders versus non-responders; however, expression of individual genes was not significant after adjustment for multiple comparisons (Supplementary Fig. 8). On gene set enrichment analysis, 252 gene sets were significantly enriched in patients with objective response (Supplementary Fig. 9). Gene sets associated with response included immune-related pathways and function such as adaptive immune response, T-cell activation, lymphocyte-mediated immunity. In the case of non-responding tumors, 88 gene sets were significantly enriched, including muscle-related pathways such as muscle cell development, striated muscle contraction, skeletal muscle adaptation (Supplementary Fig. 10).

We also observed changes in transcriptional profiles over time (comparing baseline versus week 6 on-treatment biopsy), with a general increase in immune cell signatures, particularly T-cell signatures (Supplementary Fig. 10), and a trend toward increased T-cell fraction of all nucleated cells (Supplementary Fig. 11). There was minimal overlap in highly upregulated/downregulated genes across the two treatment arms (Supplementary Figs. 12, 13).

**Fig. 6:**
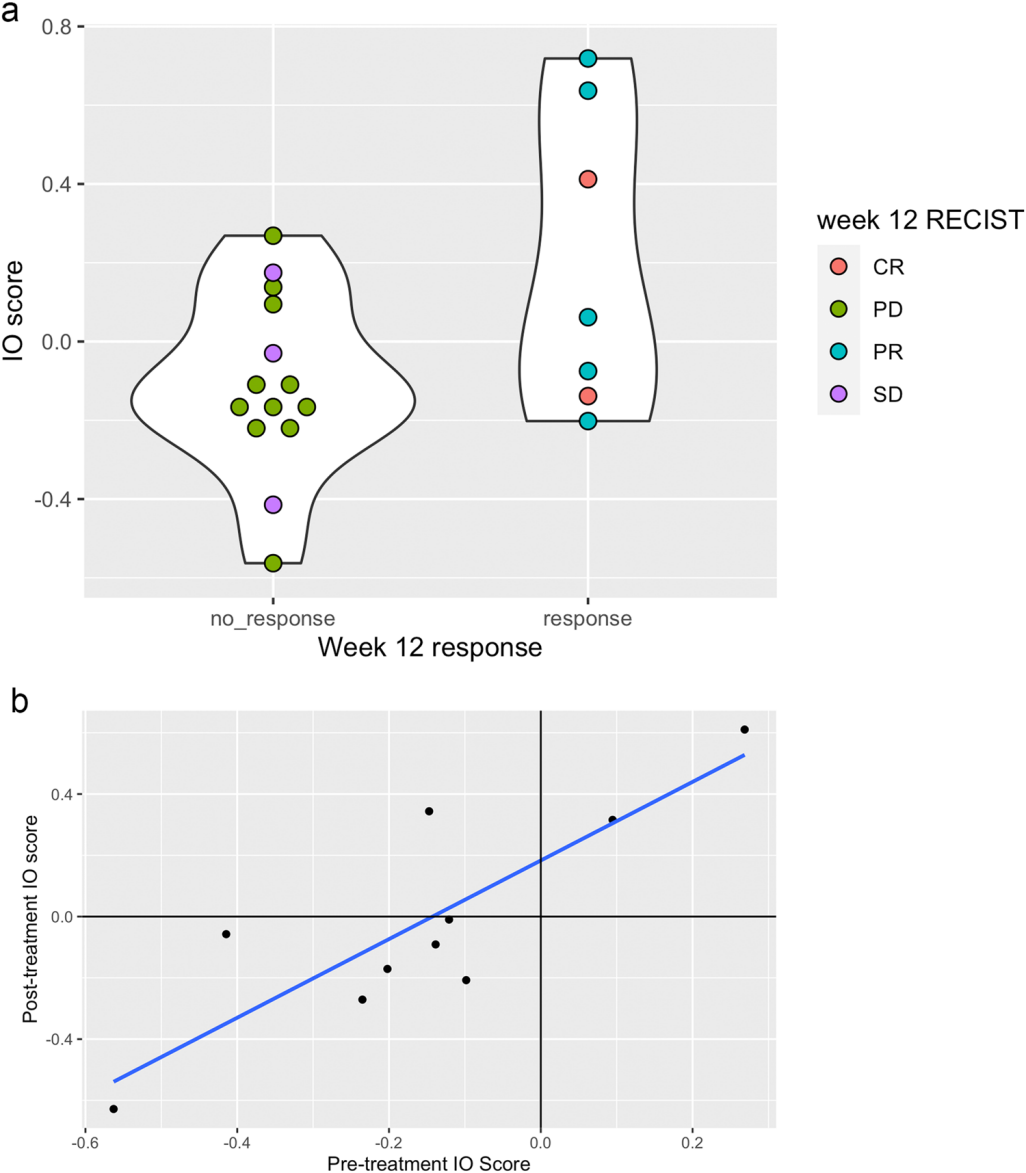
27-gene IO score as a predictive biomarker of response. **a** Distribution of 27-gene IO score according to week 12 RECIST response; **b** Correlation of pre-treatment versus on-treatment IO-score among matched biopsies. CR complete response, PR partial response, SD stable disease, PD progression of diseases.

Of special interest is the 27-gene IO score^24^, a gene expression profiling signature recently shown to predict chemoimmunotherapy benefit in the neoadjuvant TNBC setting following treatment with pembrolizumab-based chemotherapy and other immune checkpoint antibodies^25,26^. The signature is an immune classifier derived from the 101-gene TNBCtype classification system proposed by Lehmann et al.^27^. The score is reported as either a continuous variable (with higher scores indicating greater immune activation), or as a binary IO + /IO- variable. Since the signature has not yet been evaluated in the mTNBC setting, we used our dataset to conduct a preliminary analysis. IO scores were higher among patients with objective response (Fig. 6a). Using the previously described threshold for IO-score positivity, 33% of evaluable tumors were classified as IO+ (*n* = 7/21), and outcomes were favorable in this subgroup with greater ORR (IO + 43% v. IO- 29%), PFS (IO+ 162d v. IO- 83d), and OS (IO+ 687d v IO- 305 days, Fig. 6). The IO score was only weakly correlated with PD-L1 CPS score (Pearson’s *r* = 0.27), and a significant proportion of PD-L1-negative tumors were IO+ (*n* = 5/16), suggesting the two biomarkers could be complementary for identifying immune-responsive tumors. Amongst the IO + /PD-L1- subset, an ORR of 40% (*n* = 2/5, 95% CI 5–85%) was observed. ORRs in the IO-/PD-L1-, IO-/PD-L1+, and IO + PD-L1+ subsets were *n* = 2/11 (18%, 95% CI 2–52%), *n* = 2/2 (100%, 95% CI 16–100%) and *n* = 1/1 (100%, 95% CI 3–100%), respectively. Comparing matched pre-/post- specimens, IO + /IO- classification was generally concordant (Cohen’s kappa = 0.74, Pearson’s *r* = 0.84, Fig. 6b), with one case converting from IO- to IO+ following treatment. Most cases showed an increase in IO-score with treatment (*n* = 7/10 cases).

#### Multispectral immunofluorescence

We leveraged mIF to quantify the densities of various immune cell phenotypic subtypes in the tumor. Associations of immune cell density (i.e., immune cell count per unit area) and response are summarized in Supplementary Fig. 13, with ORR stratified according to whether immune cell density was above or below the median. The general trend was that increased immune cell densities correlated with clinically responding tumors (Supplementary Figs. 15–17).

**Fig. 7:**
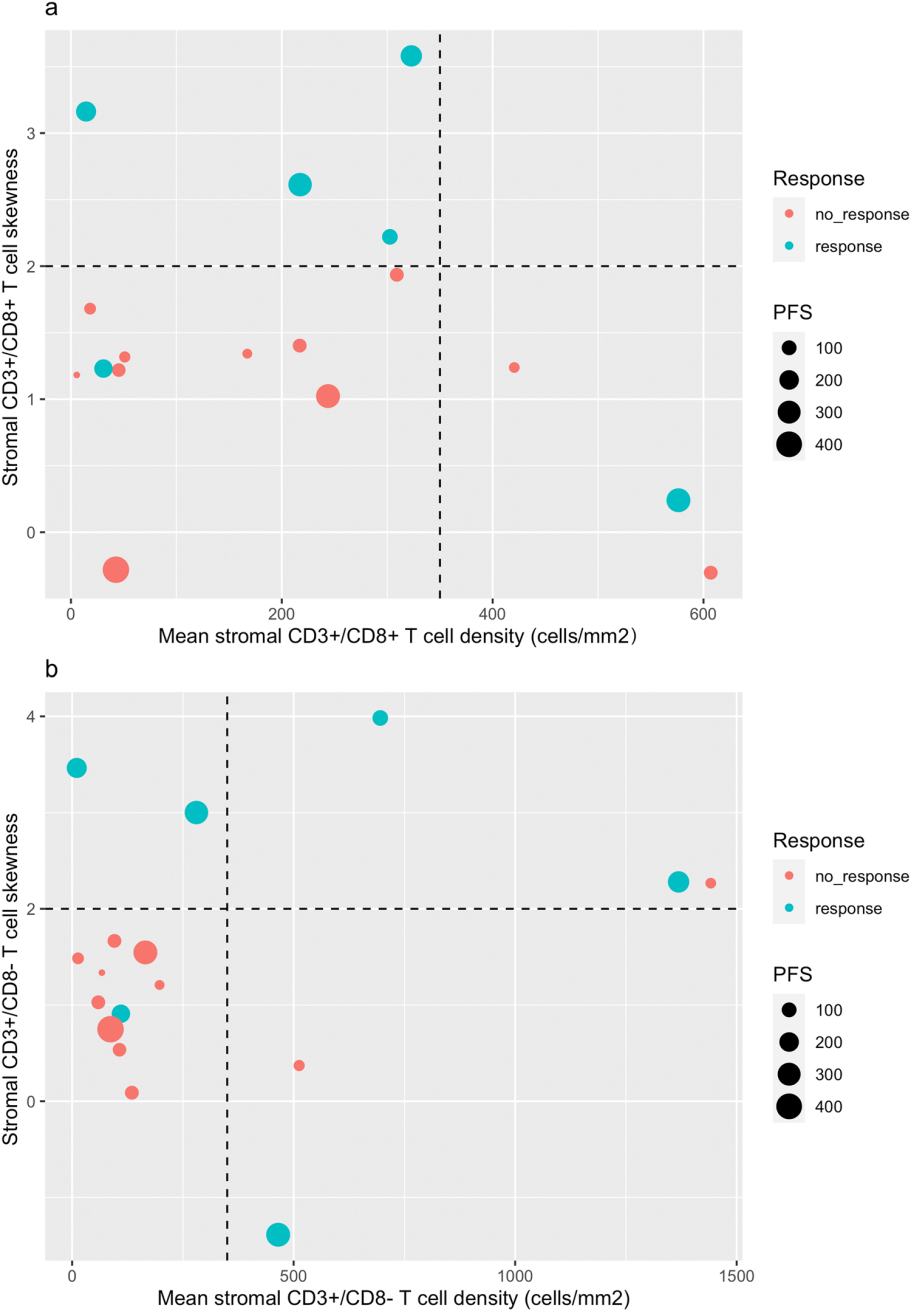
Associations of response with stromal T cell density and/or skewness. The *X* axis reports cellular density and the Y axis reports skewness scores, with high scores indicating hotspot regions within the tumor. Using 350 cells/mm^2^ density cutoff and skew = 2 cutoff, responses and PFS are enriched among tumors with high T cell density and/or T cell skew (outer & upper quadrants). **a** CD3 + CD8 + T cells; **b** CD3 + CD8- T cells. PFS progression-free survival.

Using mIF, immune cell densities can be interrogated across multiple microscopic high-powered fields (HPF, each measuring 0.36 mm^2^), affording an opportunity to evaluate the spatial distribution of immune cells as a biomarker of response. We observed that some tumors, despite having modest overall immune cell density, contained “hotspot” HPFs with higher-than-average immune cell density. The presence of hotspots within a tumor has been described as a favorable prognostic marker in melanoma and colon cancer^28,29^. To characterize tumors according to their relative abundance of hotspots, we employed a statistical metric called skewness, which summarizes the degree of spatial asymmetry of immune cell densities relative to the sample’s mean immune cell density. Tumors with hotspot-rich tumors would exhibit high/positive skewness values, whereas tumors with uniform immune cell density would have skewness values close to zero. Figure 7 illustrates the association of stromal T-cell density, skewness, and clinical response (week 12 objective response and PFS). We observed a trend of objective responses and prolonged PFS amongst tumors with either high overall immune cell density, and/or high tumor skewness. To illustrate this potential association, we defined high skewness as >2 and high immune cell density as >350 mm^2^ as a cutoff, and with these cutoffs we observed an enrichment of objective responses in the upper quadrants (high skewness) and outer quadrants (high density), relative to the lower inner quadrant (low density/low skewness).

#### Impact of therapy on peripheral blood immune cells over time

We employed mixed effects longitudinal modeling to evaluate for potential differences in peripheral immune cell counts following treatment with the two regimens. Results are summarized in Table 3 and Supplementary Fig. 18. Both regimens were associated with significant depletion of absolute leukocyte counts across 12 weeks of treatment, with the greatest impact being on B-cells and CD4 cells. Depletion was similar for both treatment regimens, with no statistical differences in the estimated effect of therapy on cell counts of the measured cellular subsets (Table 3).

**Table 3:** Peripheral blood immune cell decay related to treatment.

	Change in cell concentration over time, 10^6^ cells/L/week (95% CI)	
Cell subset	Pembro + Cape	Pembro + Paclitaxel
CD45	−27.44 (−40.77, −14.11)	−23.41 (−39.09, −7.73)
CD19	−7.15 (−10.82, −3.48)	−10.87 (−15.89, −5.85)
CD4	−14.71 (−23.68, −5.74)	−9.95 (−20.37, 0.47)
CD8	−4.33 (−7.48, −1.19)	−2.04 (−5.70, 1.62)
CD4 naïve	−6.86 (−11.99, −1.74)	−5.24 (−11.2, 0.73)
CD4 CM	−5.32 (−8.47, −2.17)	−3.24 (−6.91, 0.42)
CD4 EM	−1.96 (−3.82, −0.09)	−1.82 (−3.87, 0.24)
CD4 EMRA	−0.15 (−0.33, 0.03)	−0.11 (−0.32, 0.1)
CD4 Treg	−7.03 (−14.55, 0.49)	−2.96 (−14.28, 8.36)
CD8 naïve	−1.21 (−2.11, −0.30)	−0.19 (−1.24, 0.87)
CD8 CM	−0.65 (−1.25, −0.4)	−0.26 (−0.96, 0.44)
CD8 EM	−1.91 (−3.5, −0.32)	−0.98 (−2.82, 0.87)
CD8 EMRA	−0.60 (−1.81, 0.6)	−0.67 (−2.07, 0.74)

Linear estimates are generated using a mixed-effects longitudinal model. Rates of lymphodepletion are similar for all cellular subsets across the two therapeutic arms. Cape capecitabine, Treg T regulatory cell, CM central memory, EM effector memory, EMRA effector memory re-expressing CD45RA+.

Chemotherapy may have immunostimulatory and immunosuppressive effects. Despite a global contraction of leukocytes observed by our flow cytometry, we hypothesized that chemoimmunotherapy could stimulate antigen-specific T cell subsets and result in their expansion. To evaluate this hypothesis, we employed PBMC TCR sequencing, which facilitates the monitoring of concentrations of individual T cell clones, each which has a unique TCR sequence and unique antigen reactivity. There was no obvious trend in changes in the global T-cell diversity metrics after treatment with either regimen (i.e., T-cell richness or clonality, Supplementary Fig. 11)^19^. To investigate the dynamics of individual T cell clones, we employed differential abundance statistical testing comparing baseline versus cycle 3 timepoints^19^. Using this method, both regimens were associated with similar instances of T cell clonal expansion and contraction, with no statistical difference between the two treatment arms. Participants with above-median numbers of expanding clones had an ORR of 13%, whereas the ORR of participants with below-median numbers of expanding clones was 25%. We tracked the proportion of clonal space occupied by the top 10 clonotypes of each subject. The cumulative productive frequency of the 10 most abundant clones numerically declined over time in both treatment arms, but this was not statistically significant (Supplementary Fig. 11). Finally, we employed mixed effects logistic regression models to characterize losses or gains of T-cell clones over four serial timepoints across the two treatment groups. The results of these analyses were published previously^19^. In summary, the two regimens resulted in similar rates of T-cell clonal attrition over time, whereas pembrolizumab/paclitaxel exhibited a greater rate of emergence of new clones compared to pembrolizumab/capecitabine (odds ratio 0.455, referent pembrolizumab/paclitaxel, 95% CI 0.43–0.48)^19^.

## Discussion

Our data support a growing number of clinical trials that establish the safety and efficacy of chemoimmunotherapy in women with TNBC. Pembrolizumab plus either capecitabine or weekly paclitaxel is safe and clinically active in metastatic TNBC. Treatment discontinuation rates due to toxicity were similar to those observed with capecitabine^30^ or paclitaxel monotherapy^31^, and consistent with the low rates of immune-related adverse events observed in phase III chemoimmunotherapy trials^32–34^. There was an increased rate of diarrhea in the pembrolizumab/capecitabine arm relative to historical capecitabine controls; however, the diarrhea was effectively managed with supportive medications and dose reductions. Most subjects required dose reduction to 1500 mg BID daily, a dose commonly employed in clinical practice^35^.

Acknowledging the small sample size, we observed favorable clinical activity, particularly in the capecitabine arm, where there was an estimated ORR of 43% (95% CI: 18–71%) compared to historical controls for capecitabine monotherapy (18–30%)^36–38^. These results contrast recent phase II trial data where an ORR of 13% among 15 evaluable metastatic TNBC patients was reported, with a 6-month clinical benefit rate of 27% (compared to 43% in our trial)^10^. Notably, the majority of patients were treated in later lines (*n* = 11/16, 69%), whereas the majority of pembrolizumab/capecitabine patients were treated in the first-line setting in our trial. Moreover, in our trial, none of the patients treated beyond first-line responded, consistent with the hypothesis that chemoimmunotherapy benefit will be greater in the first-line setting. Another distinction of our trial was that it employed flat-dose 1 week on/1 week off capecitabine, which has been reported to optimize efficacy in a Norton-Simon mathematical model of tumor growth and is employed by many oncologists in clinical practice^30,39^, whereas the aforementioned study employed conventional body-surface area-based dosing, using a two week on/one week off schedule. Although the impact of capecitabine dose/schedule on response or immune effect cannot be determined with our data, we conclude that capecitabine-based chemoimmunotherapy is meritorious of further study in the first-line setting. Capecitabine plus pembrolizumab may have several advantages relative to other chemoimmunotherapy regimens. First, in patient surveys, oral chemotherapy is consistently preferred over intravenous alternatives^40^. Second, with the recent FDA approval of every 6-week pembrolizumab dosing, capecitabine plus every 6 week pembrolizumab could reduce frequency and time in infusion suites, which may improve quality of life, lower the cost of care, and reduce exposure to COVID-19. Capecitabine-based chemoimmunotherapy is also worthy of further investigation in the adjuvant setting, particularly among high-risk patients who experience suboptimal response to pre-operative chemotherapy, who are then candidates for both adjuvant pembrolizumab (per the Keynote-522 trial) and adjuvant capecitabine (per the CREATE-X trial)^13^. Phase II/III trials are ongoing to evaluate the potential role of capecitabine plus anti-PD-1/L1, including in the post-neoadjuvant setting^41,42^.

Metaplastic TNBC is a rare, aggressive, and chemo-resistant histologic subtype. We observed partial responses in two of three patients with metaplastic TNBC enrolled in the capecitabine/pembrolizumab cohort. Notably, responses occurred in these subjects despite low PD-L1 expression using the CPS > 10 biomarker threshold. The current standard of care for metaplastic TNBC is chemotherapy, with some investigators advocating for a combination of doxorubicin, bevacizumab, and everolimus/temsirolimus, which is associated with an ORR of 21%, and with substantial toxicity^43^. The clinical activity observed with pembrolizumab/capecitabine corroborates previous case reports of metaplastic mTNBC response to immune checkpoint inhibition, and warrants further investigation^44–46^. Another regimen currently evaluated in metaplastic TNBC is dual checkpoint inhibition (ipilimumab/nivolumab), which in a recent phase II study was associated with a durable response in 3/17 patients, albeit with increased immune-related toxicity^47^.

Consistent with previous reports, we observed an association of IHC PD-L1 expression with the objective response; however, we also observed objective responses amongst PD-L1-negative tumors, highlighting the imperfect nature of the IHC PD-L1 biomarker. A 27-gene IO score and CLIA-certified RT-PCR-based DetermaIO™ assay were developed as a companion gene expression profiling classifier to identify TNBC tumors with evidence of immune activation amongst the TNBC subtypes. This test has been shown to predict chemoimmunotherapy response in the neoadjuvant TNBC setting, including among PD-L1-negative tumors. In the phase III NeoTRIPaPDL1 trial, patients with IO+ tumors (*n* = 30) had a 69.8% pathologic complete response rate following chemoimmunotherapy (atezolizumab plus carboplatin/nab-paclitaxel), versus a pCR of 46.9% for IO- (*n* = 23)^25,48^. Among PD-L1-negative tumors, the difference in pCR was more pronounced (pCR: IO+75%, IO- 31%). In a similar NeoPACT trial evaluating pembrolizumab plus chemotherapy (carboplatin/docetaxel), the IO score was also highly prognostic (pCR IO+: 81%, IO- 43%)^26^. Consistent with the neoadjuvant TNBC trials, we identified a preliminary association of IO-score with response and survival, and we identified clinical activity amongst PD-L1-/IO+ tumors. Interpretation of these findings is limited by the small sample size; however, the findings are the first to be reported for metastatic TNBC and justify additional evaluation of the IO score in the metastatic TNBC setting.

Consistent with previous reports, we observed an association of H&E sTIL score with objective response, however like the PD-L1 IHC assay, subjects with low H&E sTILs may also benefit from chemoimmunotherapy. As an exploratory aim, we leveraged mIF histologic imaging to evaluate whether tumors with low overall sTIL scores, but with higher degrees of TIL hotspots, could also benefit from chemoimmunotherapy. Because no gold standard exists for defining hotspots, as an alternative we characterized tumors using a common statistical metric called skewness, which quantifies the degree of asymmetry of TIL densities across HPFs. We observed a trend of increased chemoimmunotherapy response amongst tumors with either high T cell density or high skewness, as indicated by the enrichment of clinical responses in the upper/outer quadrants of Fig. 7. While illustrative, these findings must be evaluated in larger datasets and compared to other approaches, but it demonstrates the potential for spatial TIL profiling to improve clinical prediction beyond the standard approach of reporting mean sTIL density. Collaborative efforts are ongoing via the International sTILs Working Group and other organizations to identify the optimal method for characterizing heterogeneity of TILs^49,50^.

Based upon their differing mechanisms of action, capecitabine and paclitaxel may differ in their immunomodulatory effects^51,52^. We found that both chemoimmunotherapy regimens were lymphotoxic with depletion of all measured peripheral lymphocytes subsets over time. Conversely, we identified patients with clonal T-cell expansion in the peripheral blood, which could indicate antigen-specific T-cell activation; pembrolizumab/paclitaxel appeared to induce greater emergence of previously undetectable T-cell clones compared to pembrolizumab/capecitabine. These data illustrate the potential for contrasting immunologic effects of chemotherapy. We recently reported similar findings in the early stage setting, whereby neo/adjuvant chemotherapy was associated with significant and long-lasting depletion of peripheral immune cell counts, a decline in T-cell diversity, but also stimulation of T-cell clonal expansion^19^. Based upon these collective findings, we argue that the benefits of immunotherapy can be maximized in TNBC by administering it as early as possible in the course of disease, i.e., in the neoadjuvant curative setting and/or as a first-line therapy at the time of metastatic recurrence, before patients incur the sustained and dose-dependent lymphodepleting effects of chemotherapy.

We acknowledge the limitations inherent to the design of our trial. First, the trial was not randomized, nor was it powered to formally compare clinical activity or immune effects between the two arms. For example, lower responses in the paclitaxel/pembrolizumab arm may have been explained by imbalances in confounding clinical factors, such as liver metastases, line of therapy, or distant disease-free interval. Therefore, the comparative exploratory observations are not definitive and should be used only for hypothesis generation. Randomized trials are needed to characterize definitively the impact of chemotherapy backbone on chemoimmunotherapy response, as chemotherapy selection can introduce confounding factors that may influence the immune profile. Second, we report the safety of pembrolizumab and capecitabine using the 7 days on/7 days off flat-dose schedule proposed by Norton et al., which is a widely-adopted alternative to the conventional dosing/schedule^30,39^. Differences in clinical outcome or immune profile related to dosing/schedule remain to be elucidated. Third, PD-L1 CPS was scored using SP264 (our institution’s standard at the time), which has conflicting reports of concordance with the 22c3 companion diagnostic assay^53^. Finally, because adjuvant capecitabine is increasingly utilized in the curative setting, the efficacy of pembrolizumab/capecitabine in the first-line metastatic setting must be evaluated in patients who experience metastatic progression following adjuvant capecitabine.

In conclusion, pembrolizumab plus paclitaxel or capecitabine is safe as first or second-line therapy in metastatic TNBC, with encouraging activity observed in the first-line setting, and among a subset of metaplastic TNBC patients receiving capecitabine/pembrolizumab, however, in this dataset, it is not clinically effective as a second-line therapy. Nonspecific lymphodepletion is the prevalent immunologic effect observed in the peripheral blood, as evidenced by a reduction in leukocyte counts across the spectrum of cell subtypes regardless of chemotherapy backbone, however, we observed differences in T-cell clonal expansion events that could reflect clonal T-cell immune responses related to the immunogenic effects of chemotherapy. These data support the principle that chemotherapy can be immunogenic despite being lymphotoxic in the long run and that therapeutic benefit of chemo-immunotherapy might be optimized when given in an earlier line setting. Finally, we highlight several candidate biomarkers from our exploratory analysis, such as the 27-gene IO score and TIL skewness, that merit further investigation in larger datasets.

## Methods

### Trial design

This phase Ib trial evaluated the safety of paclitaxel or capecitabine in combination with pembrolizumab. Patients were non-randomly assigned to receive chemotherapy per treating physician’s discretion. The primary objective was to assess the safety and tolerability of both combinations. Safety/tolerability was defined as receipt of at least 2 cycles of pembrolizumab plus chemotherapy without a grade 3/4 adverse event requiring discontinuation or a dose delay of ≥21 days. The secondary outcome was clinical efficacy, as measured by objective response rate (ORR) at 12 weeks based on the Response Evaluation Criteria in Solid Tumors version 1.1 (RECISTv1.1). Exploratory outcomes included additional efficacy endpoints (survival, 24-week response) and peripheral blood/tumoral immunologic biomarker assessment.

### Trial oversight

The trial was approved by the institutional review boards at Providence Cancer Institute (Portland, OR) and Cedars Sinai Medical Center (Los Angeles, CA), and was overseen by Providence Cancer Institute and the Earle A. Chiles Research Institute (Portland, OR). Written informed consent was obtained from all participants. Merck Sharp & Dohme Corp., a subsidiary of Merck & Co., Inc., Kenilworth, NJ, USA provided drug and financial support for the study.

### Screening and randomization

The study was planned for 14 subjects in each arm (paclitaxel, capecitabine). Inclusion criteria were: ER/PR negative (IHC < 1%) and HER2 negative (IHC 0-1 or IHC 2 with ISH HER2/CEP17 < 2) confirmed in metastatic biopsy; measurable disease by RECISTv1.1; ECOG 0-1; and investigator-determined indication for paclitaxel or capecitabine chemotherapy in the 1st or 2nd line setting. Exclusion criteria included: known immunodeficiency, receipt of systemic steroids or other immunosuppressive treatment within the prior 7 days, known active tuberculosis, antibody anti-neoplastic treatment within the prior 4 weeks (except denosumab), receipt of chemotherapy/radiation therapy/small molecule therapy within the prior 2 weeks, known additional cancers requiring treatment in the last 5 years, or prior treatment with an anti-PD-1/PD-L1 agent. Known CNS disease was allowed if it had been previously treated and there was no evidence of tumor progression in the 4 weeks prior to treatment. Subjects were recruited from local oncology clinics at both enrolling facilities. Emails were sent to regional clinic oncologists to ensure broad access to the clinical trials across various demographic groups.

### Trial procedures

Patients received pembrolizumab 200 mg intravenously (IV) on day 1 of each 3-week cycle and were assigned to either concurrent paclitaxel 80 mg/m^2^ IV weekly on days 1, 8, 15, of each 3-week cycle, or capecitabine 2000 mg by mouth twice daily on days 1–7 every other week. Subjects in the paclitaxel arm received dexamethasone pre-treatment per institutional policy but were encouraged per investigator discretion to reduce and/or discontinue corticosteroids following dose 2 if they had no signs of allergic hypersensitivity. Chemotherapy dose reductions were permitted. Chemotherapy dose re-escalation was not permitted.

Safety was evaluated by physical exam and using the Common Terminology Criteria for Adverse Events version 4.0 (CTCAE4.0) every three weeks, corresponding with pembrolizumab infusions. Subjects were followed for at least 18 weeks from the start of therapy to evaluate for delayed toxicities.

Efficacy was evaluated by computed tomography during screening, after 4 cycles of pembrolizumab, and every 12 weeks thereafter. ORR was assessed using RECISTv1.1^54^. PFS and OS were estimated using the Kaplan-Meier method.

### Peripheral blood immune monitoring

PBMCs were collected serially in heparinized tubes (subjects enrolled at EACRI) and Cytochex BCT tubes (all subjects). To avoid cellular loss and phenotypical changes due to cryopreservation, whole blood immune cells were analyzed either in real time on the day of collection (among subjects enrolled at EACRI) or within 24 h of collection (among subjects enrolled at Cedars-Sinai)^55^. A pilot experiment confirmed viability of cell count estimation using Cytochex BCT collection (with 24 h incubation) versus same-day analysis using blood from heparinized tubes (Supplementary Fig. 1). The flow cytometry method, including the gating strategy was described previously^55^. CD19 + B cells and CD4^+^ and CD8^+^ T cell subsets were measured using a BD LSRFortessa (BD Biosciences) flow cytometer with FACSDiva software, and analyzed using FlowJo Version 10. The gating strategy is summarized in Supplementary Fig. 2. Measured subsets included naïve (CD45RA^+^/CCR7^+^), central memory (CM) (CD45RA^-^/CCR7^+^), effector memory (EM) (CD45RA^-^/CCR7^-^), effector memory cell re-expressing CD45RA (EMRA) (CD45RA^+^/CCR7^-^) T cells, and CD4^+^ T regulatory (CCR4^+^, CD127^low^, CD25^high^) cells (Tregs)^56^.

Peripheral T-cell receptor (TCR) diversity was evaluated using the ImmunoSEQ™ T-cell receptor deep sequencing DNA-based assay (Adaptive Biotechnologies, Seattle, WA). PBMC DNA was extracted using the Allprep® DNA/RNA/miRNA Universal Kit (Qiagen, Cat # 80224) from an aliquot of cryopreserved PBMCs and submitted for analysis using instructions for sample packing and shipping provided by Adaptive Biotechnologies. ImmunoSEQ data was analyzed using the Analyzer software package (Adaptive Biotechnologies, Seattle, WA). For linear modeling, individual clonotype frequencies were exported using the Analyzer package and analyzed using the open-source R statistical package, “lme4,” using methods previously described by our group^19^.

### Tumor PD-L1 testing and immune profiling

In subjects with an evaluable baseline tumor biopsy, per institutional standard, tumor-infiltrating immune cells were assessed for PD-L1 expression using the Ventana PD-L1 SP264 assay and were scored by a trained pathologist using the Combined Positive Score (CPS)^57^. The CPS is defined as the total number of PD-L1-positive cells (tumor cells, lymphocytes, and macrophages) divided by the total number of viable tumor cells, multiplied by 100. A CPS ≥ 10 was considered positive, per recent data from the Keynote-355 trial indicating clinical benefit of chemo-immunotherapy in this subgroup^14^. Subsequent to this study, PD-L1 CPS testing using the 22c3 antibody was FDA-approved as a companion diagnostic biomarker for pembrolizumab in mTNBC, however additional material biopsy materials are unavailable for repeat staining using 22c3.

When feasible, immune infiltrates were characterized by multispectral immunofluorescence (mIF) using a previously validated panel of markers for nuclei (DAPI), tumor (cytokeratin), T-cells (CD3, CD8), regulatory T-cells (FOXP3), and macrophages (CD163)^58^. Briefly, 5 mm formalin-fixed paraffin-embedded (FFPE) slides were stained and microwave treated in citrate buffer pH 6.0., then incubated with CAPI and cover-slipped with VectaShield mounting media (Vector labs). Whole slides were scanned and digitized at 10× magnification (PerkinElmer Vectra 3.0) for visualization of tumor. Non-overlapping high-powered (20×, 0.36 mm^2^) regions of interest were selected across the span of viable tumor and scanned. InForm software (Perkin Elmer, package 2.4) was used according to manufacturer instructions to segment and phenotype cells into the following categories: tumor (CK+), cytotoxic T cells (CD3 + CD8+), CD8-negative T cells (CD3 + CD8-), regulatory T cells (CD3 + FOXP3+), and macrophages (CD163+). Mean PD-L1 quantitative immunofluorescence (QIF) was also measured for each cell. Cells were categorized as PD-L1 positive/negative using a QIF threshold that was previously shown to optimize concordance with visual categorization by the study pathologist^58^.

When feasible, RNA sequencing was performed. From deparaffinized FFPE tissue sections, RNA was extracted and purified using the Qiagen AllPrep DNA/RNA FFPE kit. 85 ng of input RNA was sequenced using the Illumina TruSeq RNA Exome kit and HiSeq 4000 instrument (2 × 76 reads paired-end configuration). Expression counts were quantified using salmon-v.0.11.2^59^, and differential gene expression analysis was conducted using edgeR^60^. Gene set enrichment analysis was conducted using the GSEAPreranked tool from GSEA 4.3.2 (reference gene sets: c2.cp.kegg.v2022.1.Hs.symbols.gmt, c2.cp.reactome.v2022.1.Hs.symbols.gmt, c5.go.bp.v2022.1.Hs.symbols.gmt, c5.go.cc.v2022.1.Hs.symbols.gmt and c5.go.mf.v2022.1.Hs.symbols.gmt)^61^. A false discovery rate of <0.25 and *p* value < 0.05 were selected as cut-off criteria.

### Statistical analysis

This trial was powered to assess safety, defined as at least 60% of subjects in each arm completing two cycles of therapy without toxicity-related discontinuation of therapy or a dose delay exceeding ≥21 days. With a sample size of 14 patients per arm there was a 0.49 probability of declaring success assuming a true safety rate of 0.6, a 0.79 probability of declaring success assuming a true rate of 0.79, and a 0.96 probability of declaring success assuming a true rate of 0.9. The secondary endpoint (week 12 ORR) was to be reported as point estimates with associated 95% confidence intervals.

Exploratory immunologic endpoints are presented descriptively for hypothesis generation. Linear trends for treatment effect on peripheral immune cell density, as well as T-cell clonotype emergence/attrition, were estimated using linear mixed-effects models. All measurements were taken from distinct samples. *P* values are provided to aid in hypothesis generation and are not corrected for multiple comparisons.

### Reporting summary

Further information on research design is available in the Nature Research Reporting Summary linked to this article.

## Supplementary information

Supplemental Material

Reporting Summary

## Data Availability

Deidentified datasets generated during and/or analyzed during the current study are available from the corresponding author upon reasonable request. RNA-seq data are available via the Gene Expression Omnibus (accession number GSE225078). TCR sequencing data is available via Adaptive Biotechnologies (clients.adaptivebiotech.com/pub/page-2023-npj, 10.21417/DBP2023NPJ). Flow cytometry data is available via flowrepository.org (accession number: FR-FCM-Z638).
