# Supplementary material for: A phase Ib trial of pembrolizumab plus paclitaxel or flat-dose capecitabine in 1st/2nd line metastatic triple-negative breast cancer
Source: NPJ Breast Cancer. 2023 Jun 21;9:53. doi: 10.1038/s41523-023-00541-2 (PMC10284878; doi:10.1038/s41523-023-00541-2)
Supplement: Supplementary file 1 — Supplemental Material [file 41523_2023_541_MOESM1_ESM.pdf]

Supplementary Materials

Title:

A Phase Ib trial of pembrolizumab plus paclitaxel or flat-dose capecitabine in $1^{st}/2^{nd}$ line metastatic triple negative breast cancer

Corresponding author:

David B. Page, MD

Earle A. Chiles Research Institute / Providence Cancer Institute

4805 N.E. Glisan St, North Tower, Suite 2N87, Portland, OR 97213

Email: david.page2@providence.org

Ph: 503.215.7807; F:503-215-6841

Supplementary figure 1: Correlation of real-time flow cytometry using heparin versus cytochex tubes

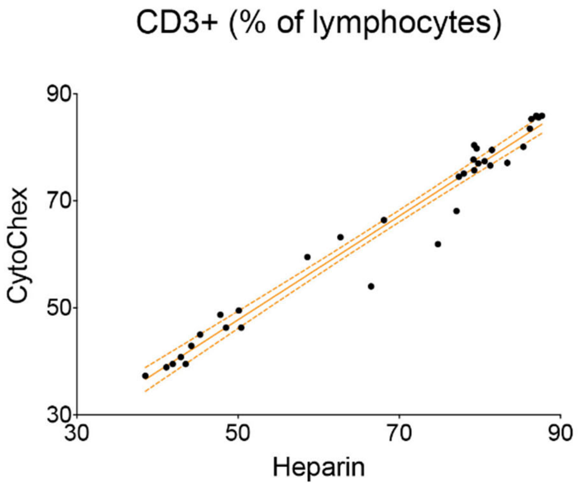

	Heparin vs. CytoChex
Spearman r	
r	0.9728
95% confidence interval	0.9453 to 0.9866
P value	
P (two-tailed)	<0.0001
P value summary	****
Exact or approximate P value?	Approximate
Significant? (alpha = 0.05)	Yes
Number of XY Pairs	35

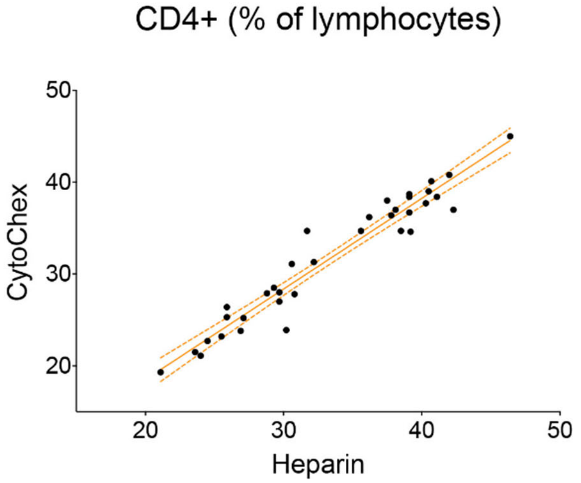

	Heparin vs. CytoChex
Spearman r	
r	0.9382
95% confidence interval	0.8778 to 0.9692
P value	
P (two-tailed)	<0.0001
P value summary	****
Exact or approximate P value?	Approximate
Significant? (alpha = 0.05)	Yes
Number of XY Pairs	35

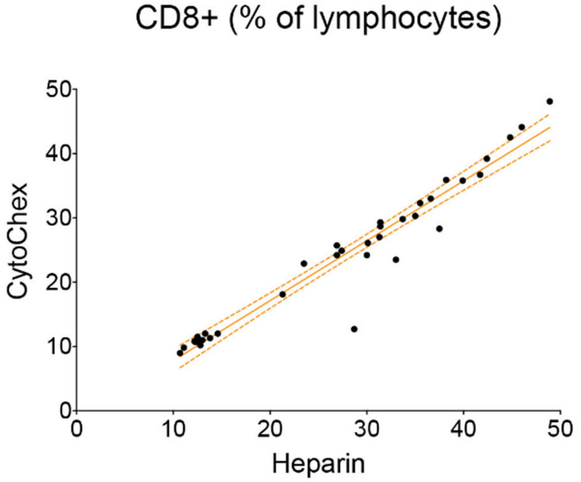

	Heparin vs. CytoChex
Spearman r	
r	0.9697
95% confidence interval	0.9392 to 0.9851
P value	
P (two-tailed)	<0.0001
P value summary	****
Exact or approximate P value?	Approximate
Significant? (alpha = 0.05)	Yes
Number of XY Pairs	35

Caption: The above correlation plots illustrate the association of cell counts using Cytochex versus heparin tubes, both in real time without cryopreservation. The solid line represents the linear trend estimate, with dotted lines being the associated 95% confidence interval. P values correspond to a 2-sided t-test of means

Supplementary Figure 2: Flow cytometry gating strategy of peripheral blood mononuclear cells

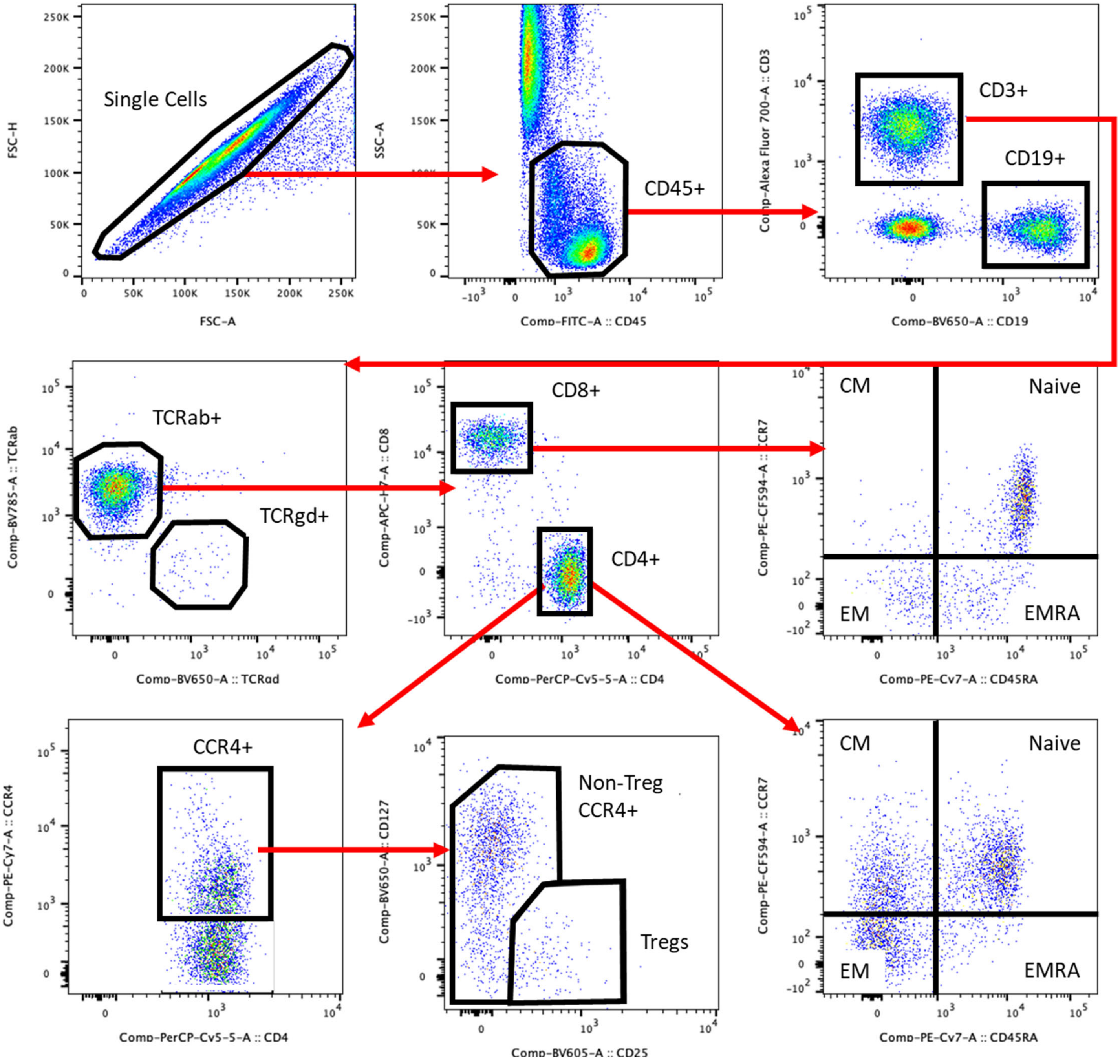

Caption: Live cells were gated on FSC/SSC (singlets) and $CD45^+$. $CD3^+$ T cells and $CD19^+$ B cells were then gated, followed by $TCRab^+$ and the indicated $CD4^+$ and $CD8^+$ T cell subsets, including naïve ($CD45RA^+/CCR7^+$), central memory (CM) ($CD45RA^-/CCR7^+$), effector memory (EM) ($CD45RA^-/CCR7^-$), effector memory cell re-expressing CD45RA (EMRA) ($CD45RA^+/CCR7^-$) T cells, and $CD4^+$ T regulatory ($CCR4^+$, $CD127^{low}$, $CD25^{high}$) cells (Tregs).

Supplementary figure 3: Influence of continuation v. cessation of dexamethasone on peripheral immune cell counts

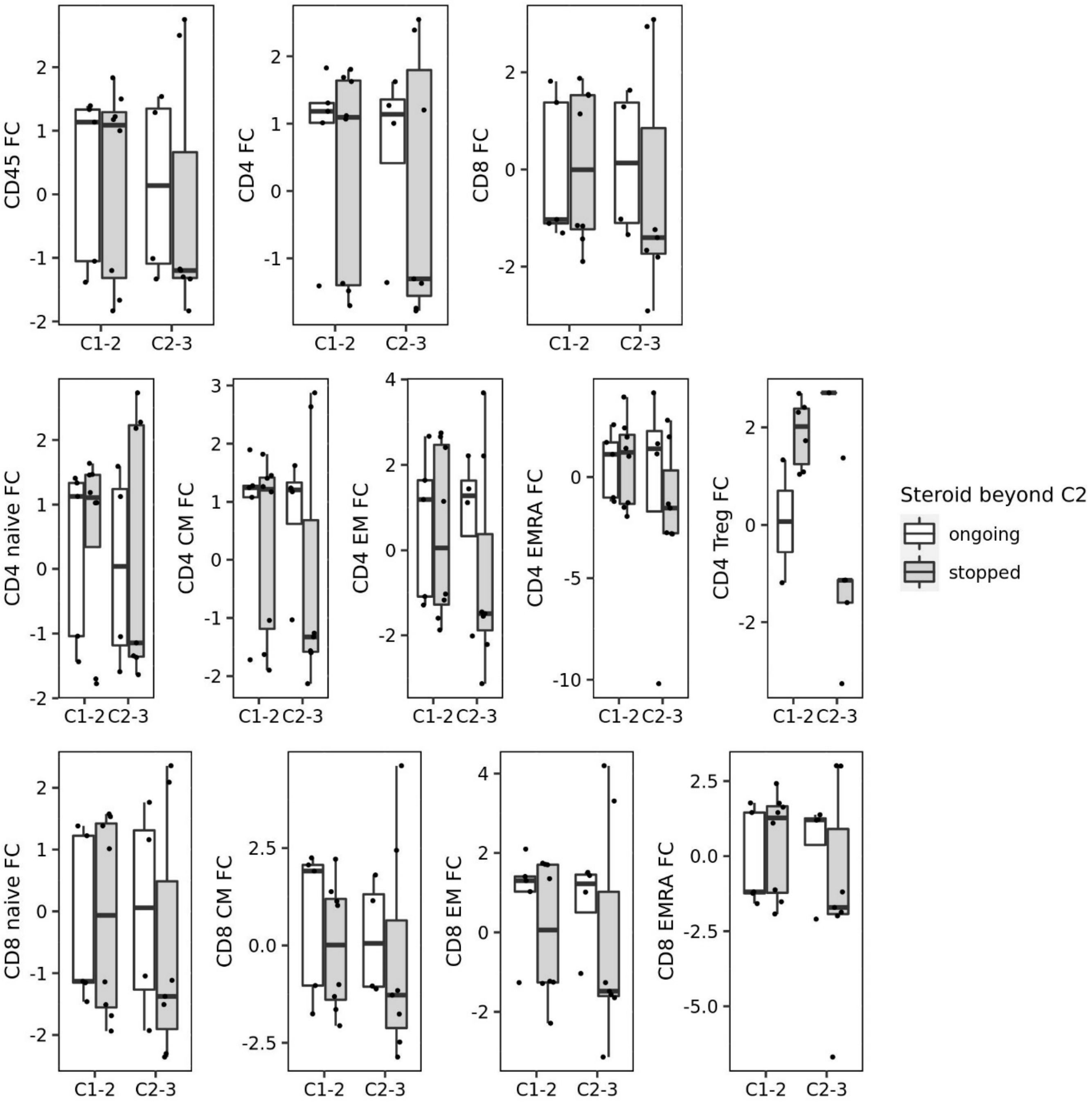

Caption: All subjects received prophylactic steroids during cycle 1 of paclitaxel, whereas some subjects stopped prophylactic steroids starting in cycle 2. Fold changes in cell counts varied significant across subjects, preventing definitive assessment of steroid effect, however the analysis does not show a consistent lymphodepleting effect of ongoing steroids.

Legend: C1-2: fold change in cell count comparing cycle 2 to cycle 1; C2-3: fold change in cell count comparing cycle 3 to cycle 2. Treg: T regulatory cell, CM: central memory; EM: effector memory; EMRA: effector memory re-expressing CD45RA+.

Supplementary table 1: Physician-reported adverse events attributed to pembrolizumab

	Patients, No. (%)	
**Adverse event**	**Capecitabine (n=14)**	**Paclitaxel (n=15)**
Any grade 1-2	6 (43%)	3 (20%)
Any grade 3-4	1 (7%)	5 (33%)
Adrenal insufficiency		
3-4	0	1 (7%)
Anemia		
3-4	0	1 (7%)
Arthralgia		
1-2	1 (7%)	0
Cough		
1-2	2 (14%)	0
Dry mouth		
1-2	1 (7%)	0
Dyspnea		
1-2	1 (7%)	0
Fatigue		
1-2	4 (29%)	2 (13%)
3-4	0	2 (13%)
Hot flashes		
1-2	0	1 (7%)
Hyperbilirubinemia		
3-4	0	1 (7%)
Hyperglycemia		
3-4	1 (7%)	0
Hypothyroidism		
1-2	3 (21%)	0
Motor neuropathy		
3-4	1 (7%)	0
Mucositis		
1-2	0	1 (7%)
Neutropenia		
1-2	0	1 (7%)
Sepsis		
3-4	0	1 (7%)

Caption: Toxicities are scored using the CTCAEv4.0. Adverse events were documented at all scheduled assessments weekly for the first 12 weeks, every 3 weeks thereafter and more frequently as clinically indicated.

Legend: CTCAEv4.0– Common Terminology Criteria for Adverse Events version 4.0

Supplementary table 2: Dose reductions for capecitabine and paclitaxel

**Dose level**	**0**	**-1**	**-2**	**-3**	**-4**
**Capecitabine**	**2000 mg BID**	**1500 mg BID**	**1300 mg BID**	**1150 mg BID**	**1000 mg BID**
% at week 4	93% (13/14)	7% (1/14)			
% at week 8	87% (11/13)		8% (1/13)	8% (1/13)	
% at week 12	40% (4/10)	40% (4/10)	10% (1/10)	10% (1/10)	
% at week 16	13% (1/8)	50% (4/8)	13% (1/8)	13% (1/8)	13% (1/8)
% at week 20		38% (3/8)	25% (2/8)	13% (1/8)	25% (2/8)
% at week 24		43% (3/7)	14% (1/7)	14% (1/7)	29% (2/7)
% at week 28		50% (3/6)	17% (1/6)	17% (1/6)	17% (1/6)
% at week 32		40% (2/5)	20% (1/5)	20% (1/5)	20% (1/5)
% at week 36		40% (2/5)	20% (1/5)	20% (1/5)	20% (1/5)
% at week 40		40% (2/4)		25% (1/4)	25% (1/4)
% at week 44		40% (2/4)		25% (1/4)	25% (1/4)
% at week 48		67% (2/3)			33% (1/3)
**Paclitaxel**	**80 mg/m^2 d1,8,15**	**80 mg/m^2 d1,8**	**70 mg/m^2 d1,8**	**60 mg/m^2 d1,8**	**50 mg/m^2 d1,8**
% at week 4	93% (14/15)				
% at week 8	90% (9/10)		10% (1/10)		
% at week 12	67% (6/9)		22% (2/9)	11% (1/9)	
% at week 16	63% (5/8)		25% (2/8)		
% at week 20	50% (2/4)	25% (1/4)	25% (1/4)		
% at week 24	33% (1/3)		33% (1/3)	33% (1/3)	

Caption: Percentages of patients receiving doses of chemotherapy, at prespecified dosing levels. Only weeks with >3 patients are listed. Dosing of capecitabine involved 7 days of twice daily administration, with 7 days off of each 14-day cycle. Dosing of paclitaxel involved infusions on days specified of each 3-week cycle.

Legend: BID – twice daily

Supplementary table 3: Guidance on management of diarrhea/colitis for combination capecitabine plus pembrolizumab, adopted from clinical protocol and study outcomes

#	**Management recommendations**
**1**	Dose interruptions may be required and are permitted according to standard practice, with clinical determination guiding whether dose modification is necessary. Dose reductions should be permanent and not re-escalated.
**2**	Subjects should be carefully monitored for signs and symptoms of enterocolitis (such as diarrhea, abdominal pain, blood or mucus in stool, with or without fever) and of bowel perforation (such as peritoneal signs and ileus).
**3**	All subjects who experience diarrhea/colitis should be advised to drink liberal quantities of clear fluids. If sufficient oral fluid intake is not feasible, fluid and electrolytes should be substituted via IV infusion.
**4**	For Grade 2 diarrhea/colitis, dosing may be held per standard practice. For diarrhea that persists greater than 3 days, administer oral corticosteroids.
**5**	For Grade 3 or 4 diarrhea/colitis that persists > 1 week, treat with intravenous steroids followed by high dose oral steroids.
**6**	For persistent grade 2 or higher diarrhea, consider GI consultation and endoscopy to confirm or rule out colitis.

Supplementary figure 4: Associations of response with peripheral blood immune biomarkers

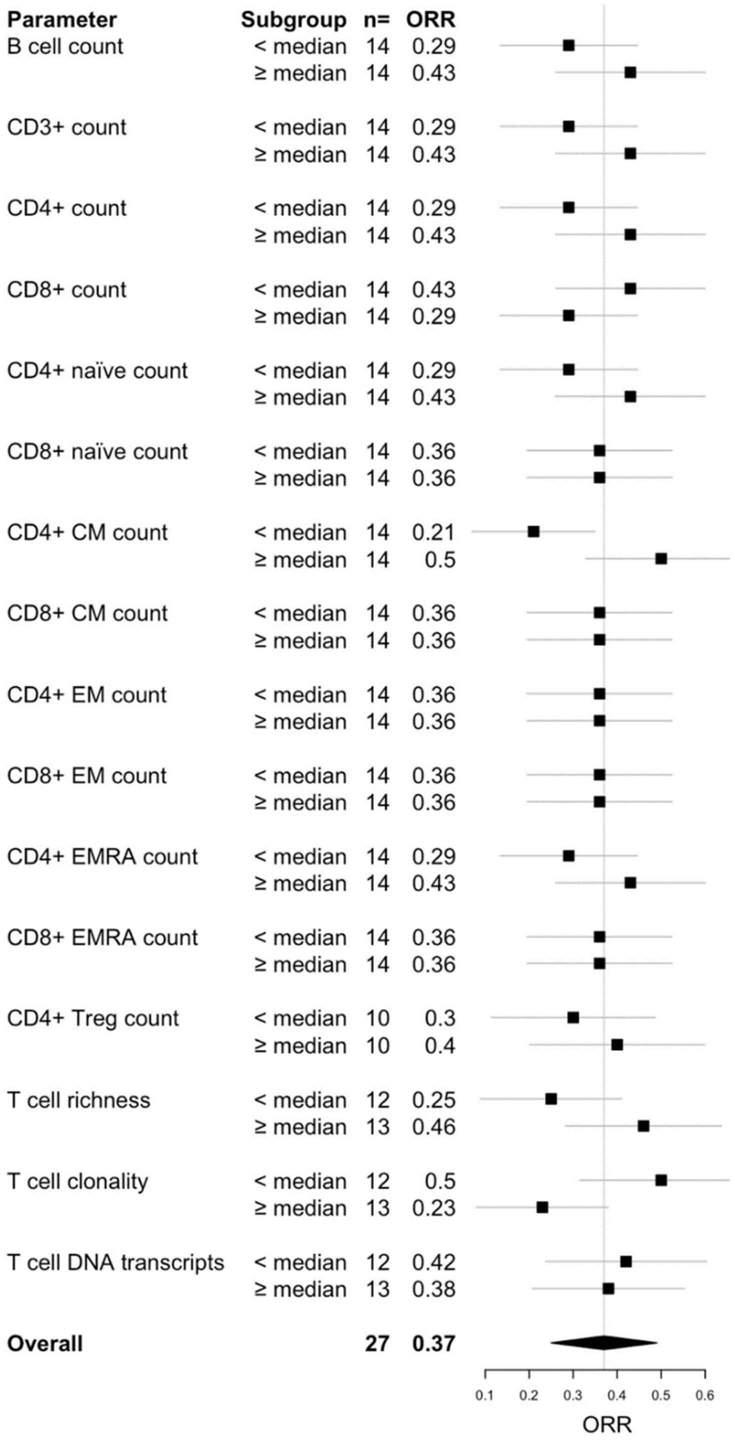

Caption: Intervals represent the 80% confidence interval of the point estimate of response rate. Statistical testing is not conducted given the exploratory nature of the assessment.

Legend: ORR: overall response rate; Treg: T regulatory cell, CM: central memory; EM: effector memory; EMRA: effector memory re-expressing CD45RA+.

Supplementary figure 5: Baseline peripheral blood biomarkers according to response

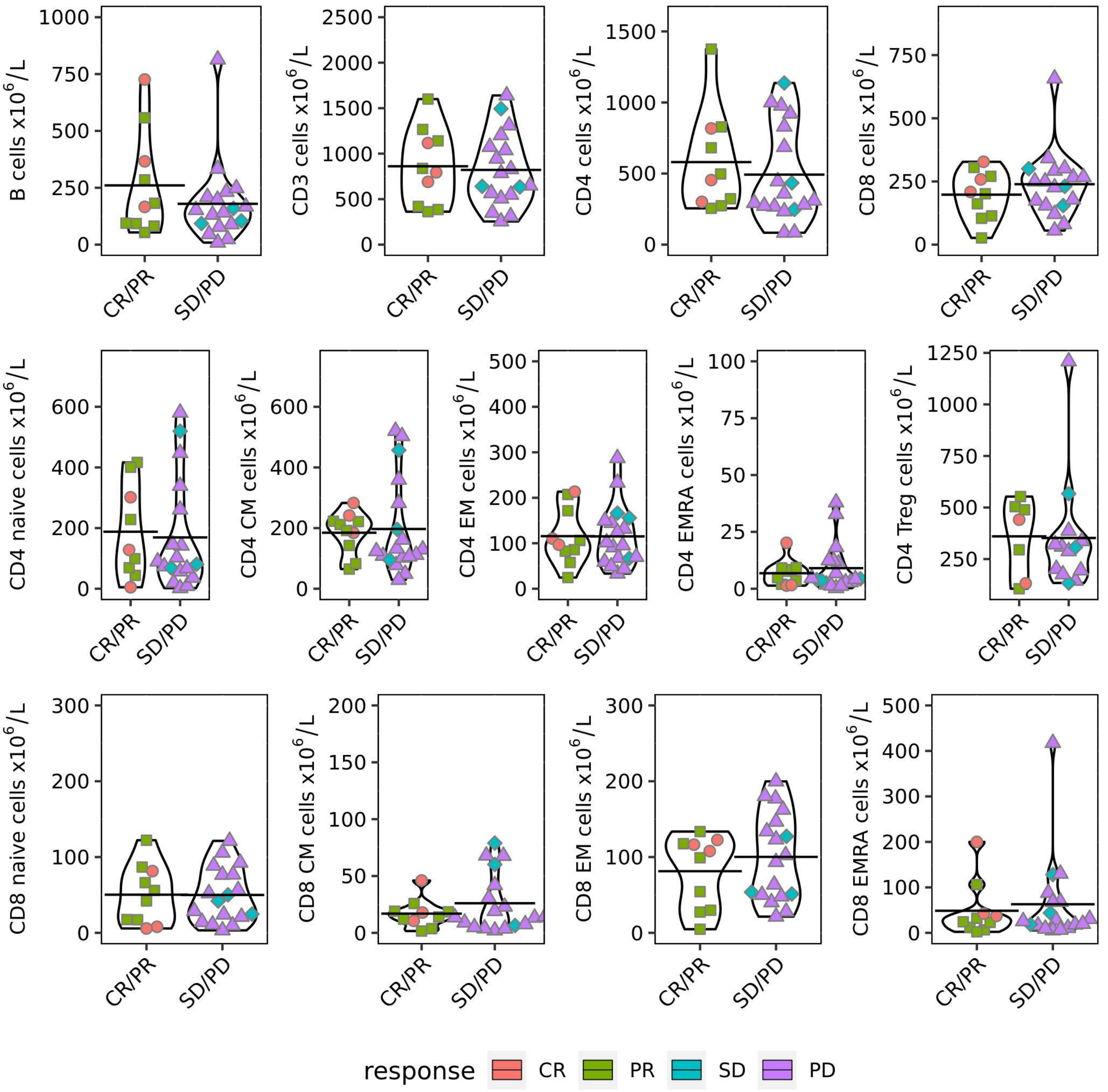

Caption: No apparent differences in peripheral baseline cell counts were observed according to response. Means are illustrated by the horizontal line.

Legend: CR: complete response; PR: partial response; SD: stable disease; PD: progression of disease; Treg: T regulatory cell, CM: central memory; EM: effector memory; EMRA: effector memory re-expressing CD45RA+.

Supplementary figure 6: Baseline peripheral T-cell diversity metrics according to response

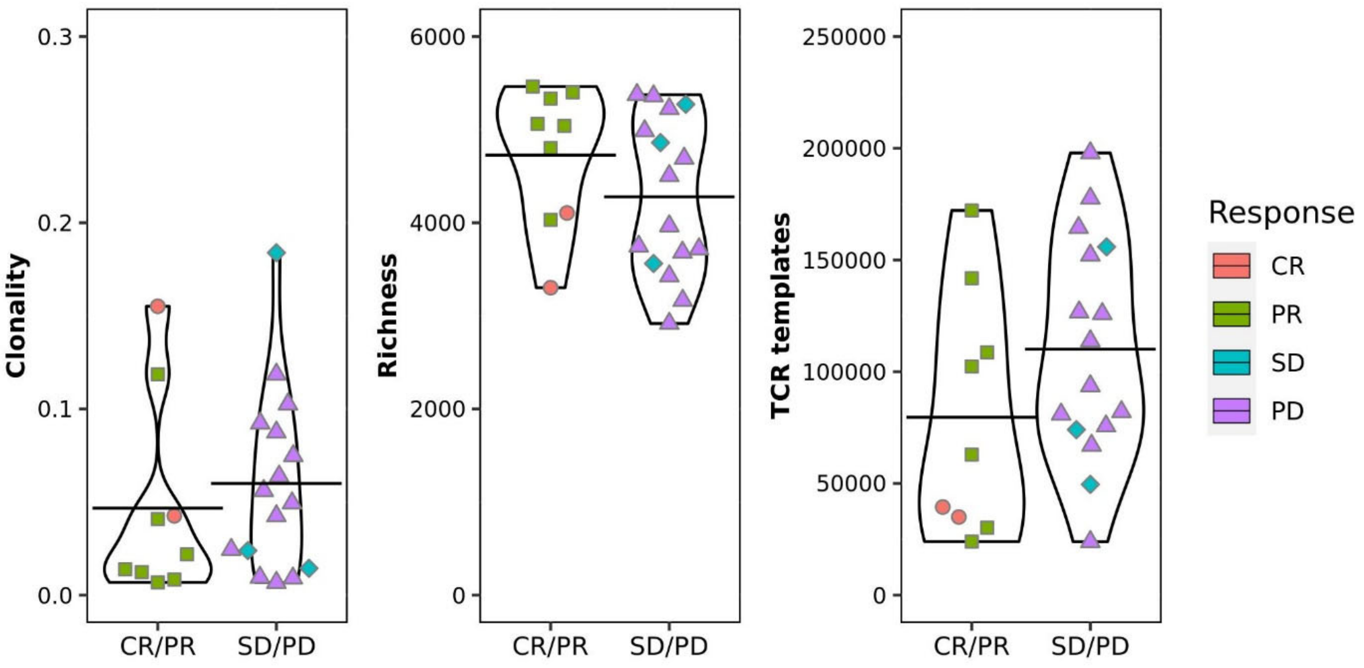

Caption: Distribution of previously-described T-cell receptor sequencing metrics according to clinical response. Means are illustrated by the horizontal line.

Legend: CR: complete response; PR: partial response; SD: stable disease; PD: progression of disease; TCR: T-cell receptor

Supplementary figure 7: Immune cell type score between patients with and without clinical benefit.

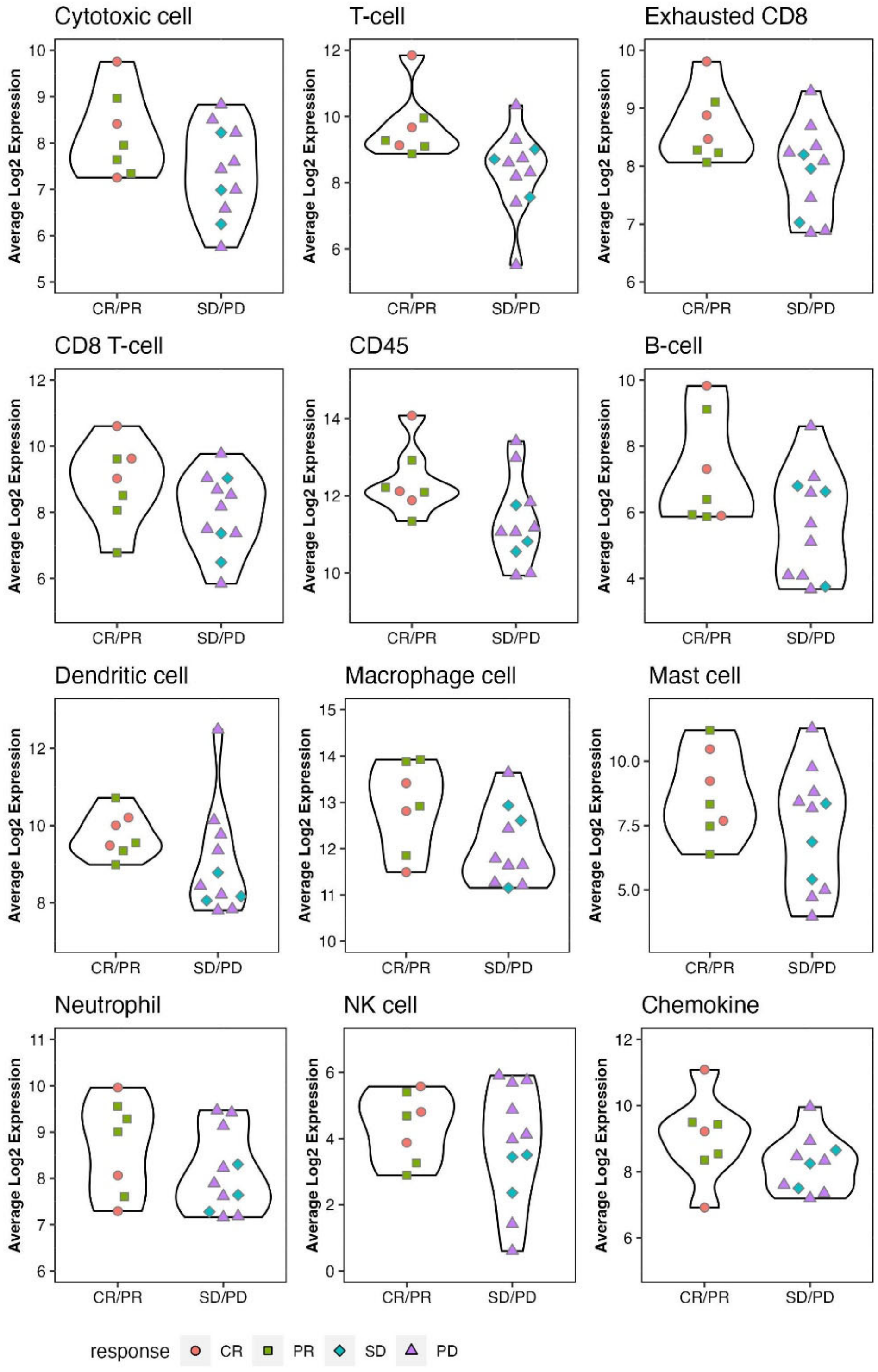

Legend: Distributions of previously described baseline RNA cell signatures according to clinical response. Genes used to compute immune cell type score is defined previously.[56] CB : clinical benefit (clinical response or stable disease), NO_CB : no clinical benefit, CR – complete response, PR – partial response, PD – progressive disease, SD – stable disease.

Supplementary figure 8: Volcano plot and table showing enriched genes among patients with clinical benefit versus not

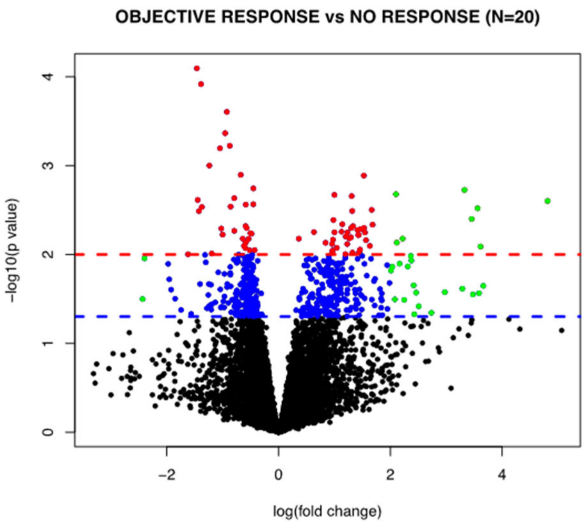

Green: >2-fold difference
Red: unadjusted p<.01
Blue: unadjusted p<.05

Upregulated Genes

Gene symbol	logFC	P.Value
ZNF623	4.813048497	0.0024941
ATP12A	3.663839303	0.0225435
CES1	3.613302658	0.0081421
CEACAM5	3.586639495	0.0272368
PSMB9	3.561129662	0.0030208
C4B	3.477219404	0.0281721
ADH1B	3.455093406	0.0039845
MS4A1	3.32635368	0.0018764
ATF6B	3.288408717	0.0242604
BAG6	2.973931527	0.0265607
TMPRSS3	2.733782292	0.0453022
COL6A5	2.50772643	0.0382149
C7	2.461655354	0.0268296
HLA-B	2.427536902	0.047053
TNXB	2.417526074	0.0222344
XPNPEP2	2.37561342	0.0117114
PTGDS	2.366890435	0.0103813
F8A3	2.312826031	0.0136435
GFRA1	2.247494941	0.0323935
FCRL3	2.21871375	0.0066261
SELE	2.164097231	0.0127053
CCL19	2.115072863	0.0073429
SIRPB1	2.100191841	0.0020997
IL33	2.081377877	0.0320079
PLAC8	2.0355918	0.0137541
COL6A6	2.011064459	0.0151726
IDO1	1.98499261	0.0208745
CACNA1G	1.946804086	0.0409863
TNN	1.942066421	0.0131658
SLITRK5	1.858064987	0.048155
CDHR3	1.846133077	0.0329142
SLC12A3	1.844676473	0.0273839
TDRD9	1.836166316	0.0397769
ADAM33	1.835806054	0.0312482
PMEL	1.815361389	0.0244973
ADAMDEC1	1.79756783	0.0481982
SELL	1.725375784	0.018814
PLIN1	1.720007343	0.0374854
BANK1	1.706000554	0.0199328
ENPP2	1.681259058	0.0046091
TDO2	1.667979883	0.0321249
CD3D	1.665615898	0.0031402
LAMP3	1.654727152	0.01116
CCDC170	1.633737003	0.0080098
CCR7	1.578380471	0.0173169
ITK	1.56397117	0.0069039
CD3E	1.544777964	0.0056409
TENM1	1.539894575	0.0311011
JAML	1.535568238	0.012729
RIPOR3	1.529665717	0.0050169
CLIC2	1.525318799	0.0012944
CD3G	1.496708275	0.0104008
SLAMF6	1.486804335	0.0416346
FCMR	1.486394213	0.0291569
CSF2RB	1.470077414	0.0051627
P2RX1	1.470017891	0.0283066
AOAH	1.458391419	0.0088
SLAMF7	1.446746824	0.0246829
ABCB1	1.442207945	0.0093784
IFIT1	1.442202394	0.0480858
CD48	1.435246747	0.006016
RASGRP2	1.424129178	0.0060355
TOX	1.423388886	0.0355438
ZAP70	1.413233253	0.0112043
CD2	1.408297676	0.0052985
IL7R	1.395001326	0.0327492
LAX1	1.387723267	0.0368488
TNF	1.386294668	0.0409045
SIGLEC10	1.37885856	0.0320218

Gene symbol	logFC	P.Value
FCN1	1.375562735	0.027332
CYP26B1	1.367426805	0.020456
GIMAP7	1.363513904	0.024807
IL2RG	1.331992059	0.004799
SPN	1.322119151	0.009528
ARHGAP15	1.317016306	0.003236
CSF2RA	1.307657471	0.002198
IKZF3	1.304177688	0.014289
IL18R1	1.302620822	0.039515
SPOCK2	1.302108889	0.017023
SLFN12L	1.29887854	0.005039
MATN2	1.29484366	0.006406
HIST1H3G	1.289132395	0.021637
CD6	1.286316814	0.021671
HRH2	1.27125899	0.042348
IFIT2	1.269435897	0.042402
ITGAL	1.26815749	0.006576
GIMAP4	1.232780504	0.005641
SIGLEC14	1.232428955	0.024844
TMC8	1.227746932	0.007628
ZBP1	1.226704623	0.04957
GIMAP5	1.226382094	0.027639
BIRC3	1.225470726	0.033397
FGD2	1.216616048	0.02015
MTUS1	1.216433496	0.042237
LCK	1.21144489	0.020933
MCOLN2	1.211204649	0.017765
IKZF1	1.200299931	0.006277
CCL5	1.195584749	0.032454
PTPRC	1.19495519	0.009903
PPP1R16B	1.193708809	0.010824
CD96	1.191598002	0.011046
IL10RA	1.171608063	0.020338
ITM2A	1.169355327	0.031746
STAT4	1.163125816	0.031552
SASH3	1.1547663	0.021239
CD247	1.143721657	0.014646
ARHGAP25	1.140617959	0.004563
RASGRP1	1.140137237	0.019677
RASAL3	1.140112431	0.012424
PDE3B	1.13506693	0.017772
BIN2	1.1261448	0.005534
GBP5	1.125984561	0.027365
PLEK	1.113702673	0.027159
GIMAP8	1.110232505	0.024278
MPEG1	1.10580309	0.018133
IL12RB1	1.092993979	0.021943
PREX2	1.086167736	0.032419
CCDC69	1.076015087	0.012936
SP140	1.070609407	0.012481
DAPP1	1.066695272	0.015073
CYP2S1	1.065977885	0.025129
DOK2	1.062766151	0.049343
TFEC	1.062621745	0.042984
CARD11	1.047792367	0.032737
IRF8	1.046823587	0.013762
VAV1	1.044163818	0.020158
MS4A6A	1.042906564	0.027518
SEPT1	1.04216318	0.016583
FGL2	1.041967636	0.008418
CD4	1.041556346	0.029825
IL21R	1.036987387	0.036565
IL2RB	1.035935687	0.027097
SAMSN1	1.015657598	0.012795
GBP4	1.014219054	0.015949
IL18	1.006428961	0.047064
PREX1	1.000301785	0.005614

Downregulated Genes

Gene symbol	logFC	P.Value
LRP2	-3.311885077	0.219017895
PRKAA2	-2.43256931	0.03170946
PPFIA4	-2.400010768	0.011063527
PHGDH	-1.976689212	0.01276982
ANGPTL4	-1.955738607	0.0190045
GSTO2	-1.922930943	0.024807518
STC1	-1.850288932	0.031456233
CBS	-1.745253983	0.042029283
SLC7A5	-1.620580152	0.00994172
MGST1	-1.563259395	0.046503136
GAPDH	-1.461888481	8.04E-05
ENO2	-1.44707756	0.002428922
ANO1	-1.424892193	0.003247748
CDH3	-1.404418868	0.021484796
P4HA1	-1.387793378	0.000121102
PLOD2	-1.37314193	0.002921357
HSPA6	-1.315767021	0.010092377
LIMCH1	-1.301727653	0.012254092
PLEKHG6	-1.281745818	0.040350355
BNIP3	-1.258750628	0.028550611
SLC2A1	-1.247874329	0.021482321
HILPDA	-1.243401586	0.037949496
DTNB	-1.239080853	0.000996433
ALDOC	-1.214047835	0.037488356
B4GALNT4	-1.208315885	0.021947776
CTAGE4	-1.203931843	0.041776655
PFKP	-1.196892808	0.009766593
GOLGA8B	-1.192490651	0.036123814
PDK3	-1.132084925	0.034587876
PFKFB4	-1.079351055	0.015897983
TPI1	-1.048140413	0.000638656
PPP2R3A	-1.04311149	0.024688851
NDRG1	-1.034335731	0.039423688
PDK1	-1.025676217	0.005106502
TLE2	-1.018298763	0.038835593

Legend: All genes with greater than 10-fold differential are listed. Genes with an estimated greater than 100-fold differential are illustrated in green. Genes that are associated with unadjusted p values <.01 or .05 are illustrated in red and blue, respectively. logFC: log fold change; P.Value: unadjusted P-values.

Supplementary figure 9: Gene set enrichment analysis

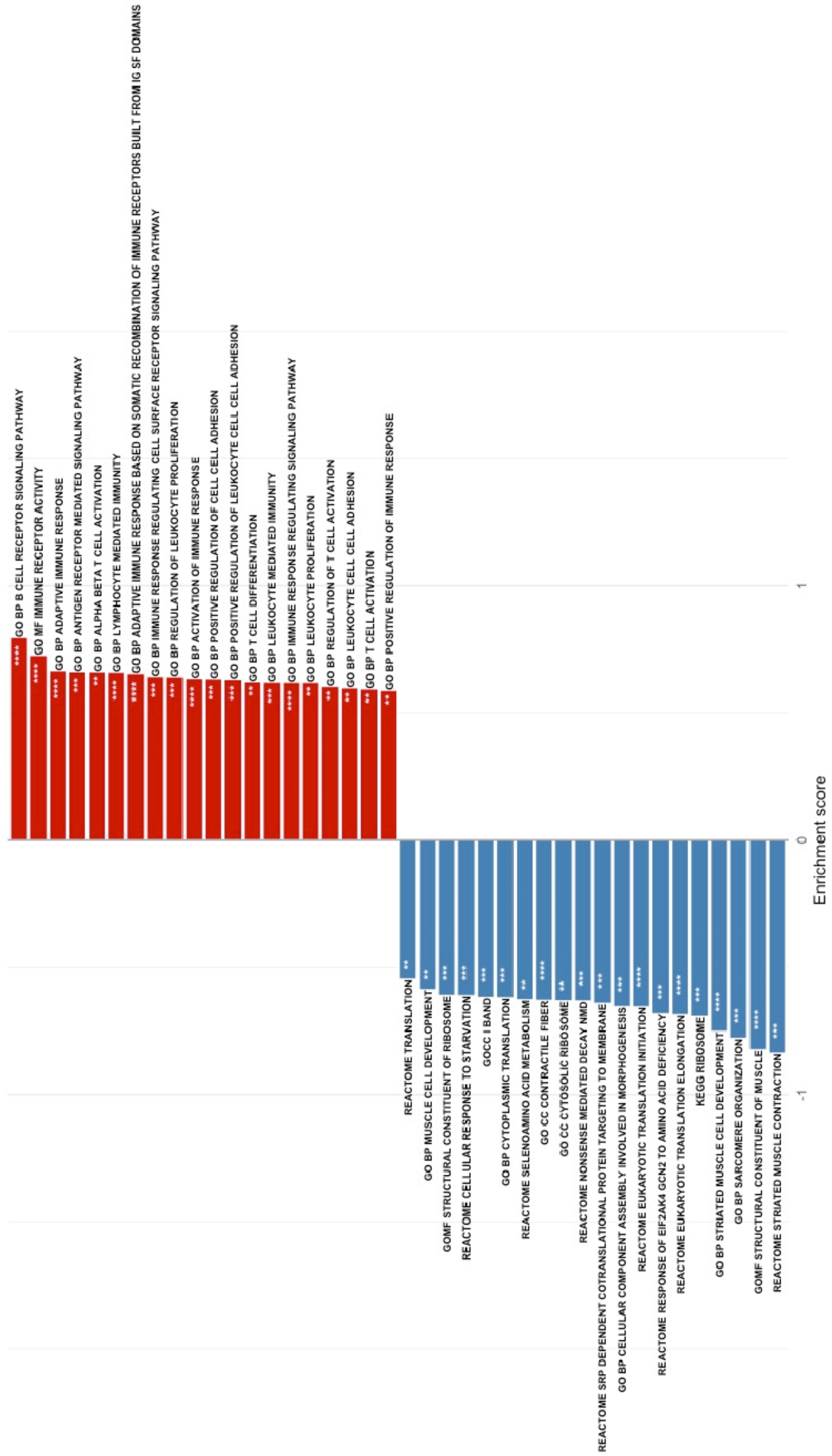

Legend: Top 20 significant gene sets enriched by genes that are up regulated in responders (red) and non-responders (blue). ****: false discovery rate (FDR) q-value<.00001; ***: FDR q-value<.0001; **: FDR q-value<.001

Supplementary figure 10: Changes in immune cell RNA signatures related to capecitabine versus paclitaxel.

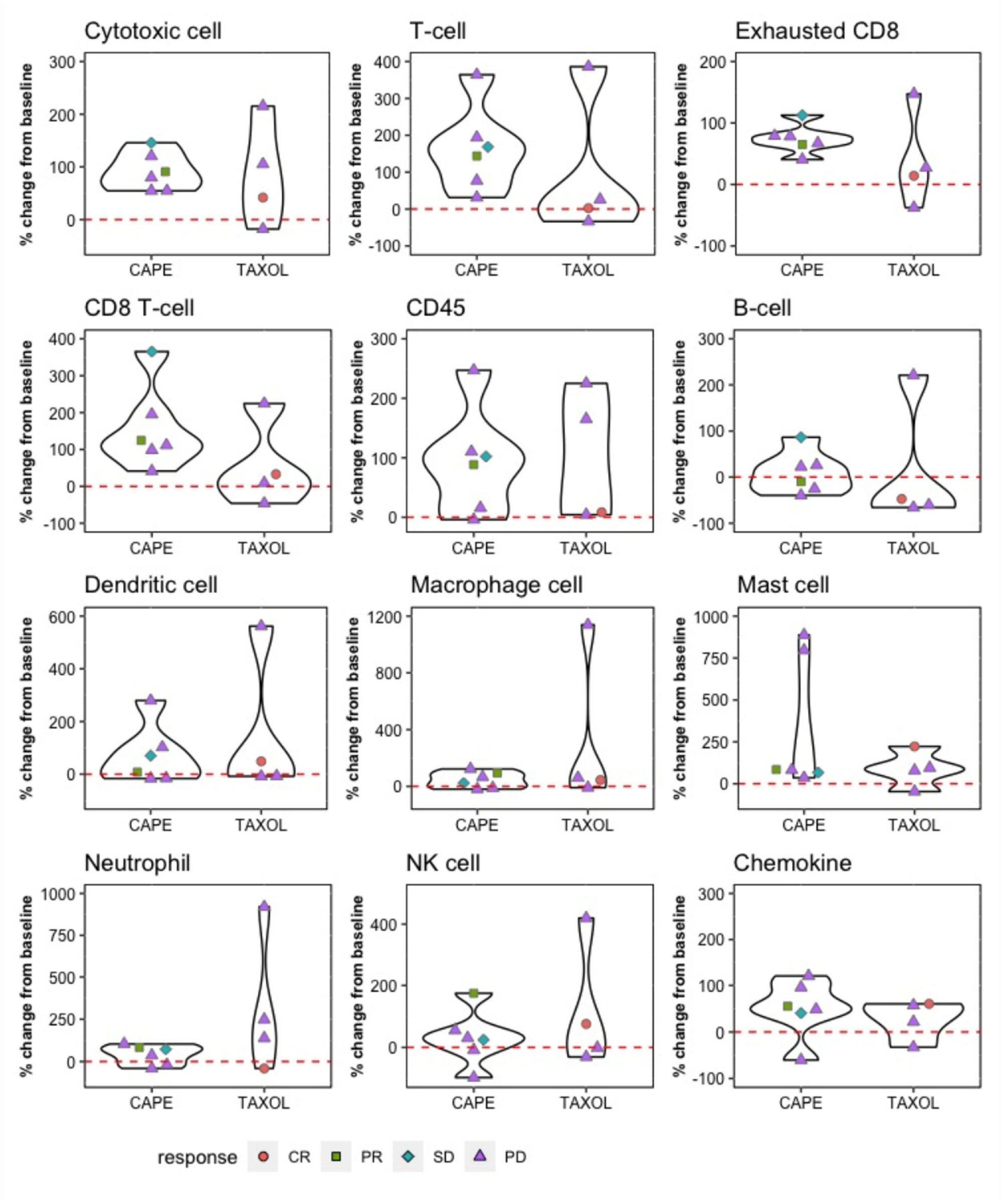

Legend: Distribution of fold-changes RNA signatures following therapy with either capecitabine or paclitaxel. Genes used to compute immune cell type score are defined previously.

Cape: capecitabine; CR – complete response, PR – partial response, PD – progressive disease, SD – stable disease

Supplementary figure 11: Effect of therapy on T cell diversity metrics

a

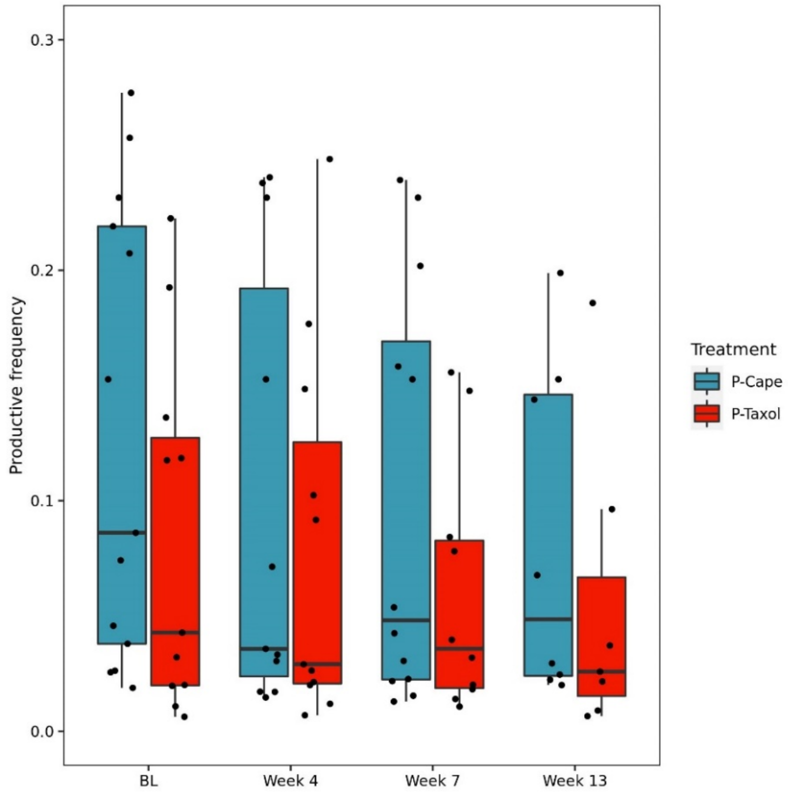

b

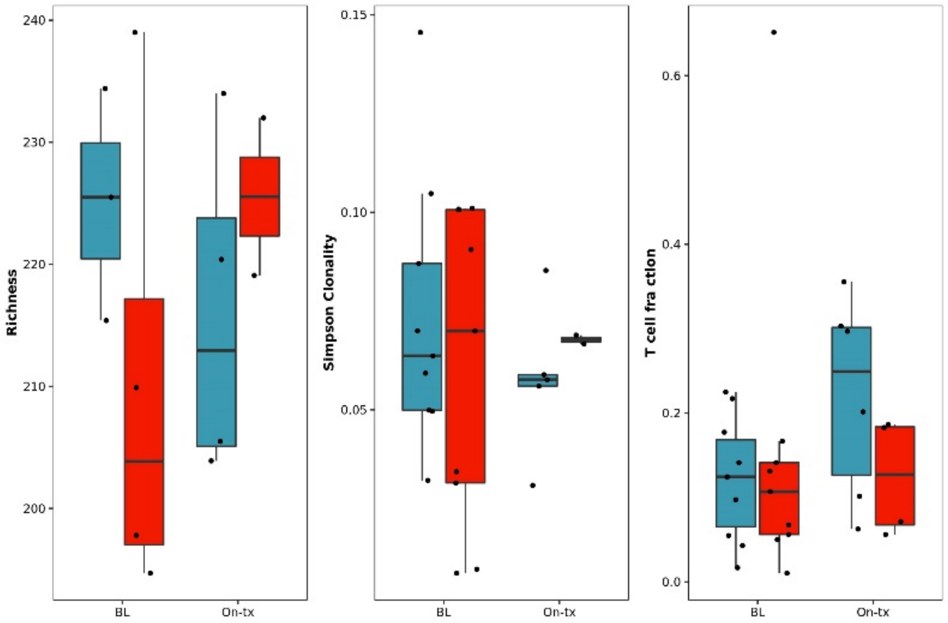

Legend: A. Change in productive frequency of top 10 clones during treatment. B. Change in T-cell diversity metrics during treatment. P-Cape: pembrolizumab + capecitabine; P-Taxol: pembrolizumab + paclitaxel; BL: baseline.

Supplementary figure 12: Volcano plot and table showing changes in genes following capecitabine/pembrolizumab

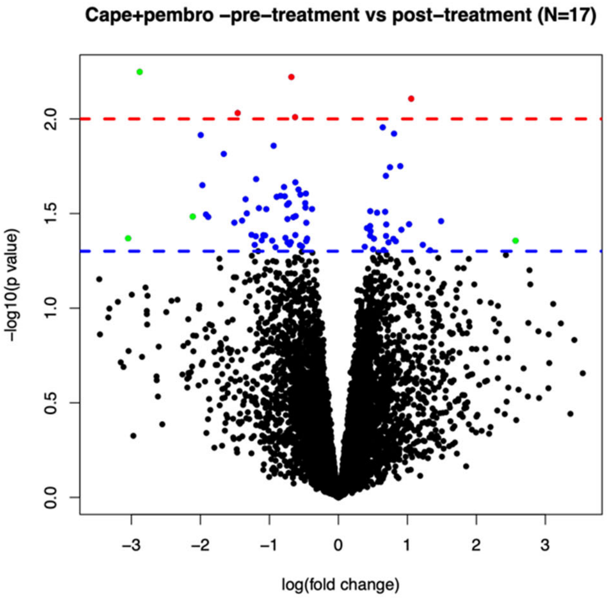

Upregulated

Gene symbol	log fold change	P.Value
DNER	2.568034762	0.04400165
ADORA2B	1.488227624	0.03469751
NOD2	1.328910059	0.04946219
TVP23B	1.22371323	0.04626969
SLC6A8	1.055838058	0.00782063
PFKFB4	1.0247326	0.03602954

Green: >2-fold difference
Red: unadjusted p<.01
Blue: unadjusted p<.05

Downregulated

Gene symbol	log fold change	P.Value
SCGB3A1	-3.048232227	0.04276144
SCN7A	-2.879792864	0.00564326
SGCE	-2.111234592	0.03282437
ADGRV1	-1.995121115	0.0121706
HLA-F	-1.97133653	0.02240301
HTRA4	-1.919416718	0.03198055
TMEM255A	-1.887203294	0.03296431
CES3	-1.6614202	0.01530858
KCNJ5	-1.507691374	0.03532331
TLR7	-1.459932105	0.00931291
ALDH1A1	-1.395700015	0.03446596
CPM	-1.345569265	0.02656635
MTARC2	-1.32742907	0.03156467
TM4SF18	-1.259373199	0.04095327
CD28	-1.212735584	0.04629563
IGSF6	-1.196334973	0.04171075
C2	-1.192639687	0.02080366
CDH6	-1.151669668	0.02961265
TBX5	-1.110923078	0.04377559
AIF1	-1.088560637	0.04114346
DDR2	-1.062454686	0.04129971
ADHFE1	-1.045325384	0.02997068

Legend: All genes with greater than 10-fold differential are listed. Genes with an estimated greater than 100-fold differential are illustrated in green. Genes that are associated with unadjusted p values <.01 or .05 are illustrated in red and blue, respectively. logFC: log fold change; P.Value: unadjusted P-values.

Supplementary figure 13: Volcano plot and table showing changes in genes following paclitaxel/pembrolizumab

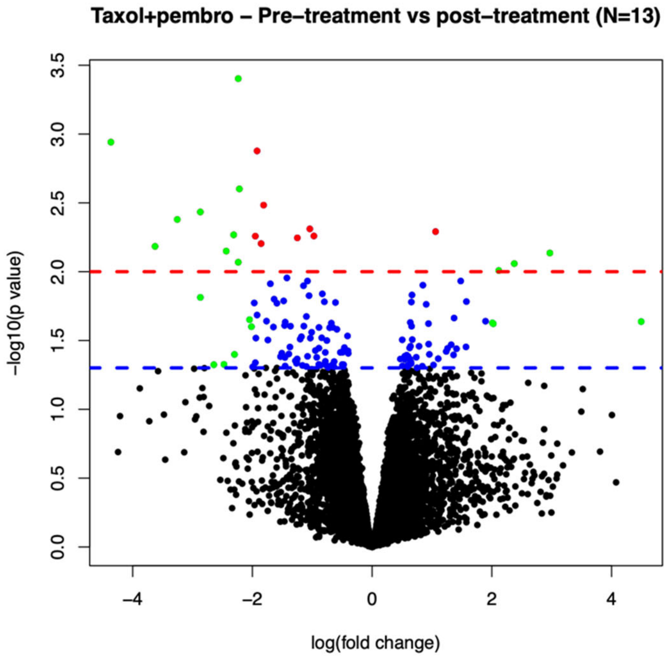

Upregulated

Gene symbol	logFC	P.Value
CCL21	4.49481831	0.02304223
SPP1	2.96911383	0.00731625
HIST1H2BL	2.37494033	0.00873522
HIST1H2BM	2.11515522	0.00980096
HIST1H2AI	2.02435133	0.02387955
HIST1H2AJ	2.00936368	0.0235438
CPNE7	1.89452451	0.0228919
RNF207	1.5748249	0.01648905
C1QTNF5	1.56774418	0.03519731
SULF1	1.47984882	0.01168809
SKIV2L	1.40983349	0.03630797
MINDY1	1.36925123	0.02170867
SLC12A8	1.36034974	0.04023951
ZDHHC8	1.32095034	0.03391518
HAGHL	1.25969821	0.03608887
PRKX	1.23792387	0.03797141
MANEA	1.10401222	0.04286419
VARS	1.05956174	0.00511319
DNPH1	1.02500816	0.04763352

Green: >2-fold difference

Red: unadjusted p<.01

Blue: unadjusted p<.05

Downregulated

Gene symbol	logFC	P.Value
C12orf56	-4.364125004	0.001144232
FAM3B	-3.626146426	0.006546511
HCAR3	-3.255059512	0.004172703
HCAR2	-2.869998778	0.003682009
PTGS2	-2.869765614	0.015373573
ALG1L	-2.644361047	0.047407702
DYNC2H1	-2.475363832	0.047155548
IL1R2	-2.437821688	0.007082009
NCAM2	-2.313115434	0.005398152
SPAG17	-2.299305848	0.039921776
SNCAIP	-2.236985934	0.000395596
DACH1	-2.236482842	0.008535097
ENTPD3	-2.218045529	0.002502937
FLT3	-2.047598117	0.02231397
SELE	-2.016394536	0.025099673
FBXW10	-1.995390531	0.049160925
OSR2	-1.969658591	0.016873068
CA12	-1.951332551	0.005519737
HAL	-1.951235185	0.045833567
FANK1	-1.941986188	0.030356912
TENM2	-1.922535121	0.001325801
JAKMIP3	-1.92219717	0.020647926
C2CD6	-1.854865502	0.006249075
TNFRSF10C	-1.813016876	0.003283953
WDR63	-1.764757095	0.022865335
TDRP	-1.741605377	0.031361976
MEFV	-1.700336976	0.012239201
ZNF596	-1.649021798	0.025245759
IL1RN	-1.637066811	0.015828259
AAMDC	-1.591273431	0.01694542
HLA-DMA	-1.530727074	0.041423224
TEX9	-1.508605821	0.039091419
EPHA4	-1.477943481	0.016305517
SEMA6D	-1.462210815	0.024585375
EID3	-1.456845409	0.042011248
HPX	-1.453434894	0.048429488
LURAP1L	-1.452514639	0.023229696
OGN	-1.424185845	0.011102569
C12orf66	-1.391225386	0.039617166
IFIT2	-1.373159192	0.035253303
SHROOM2	-1.363912124	0.024881029
FBLN1	-1.281877203	0.044394503
FMO4	-1.263173477	0.046308529
TTLL7	-1.259222559	0.045232032
COL7A1	-1.252606063	0.043011141
MXD1	-1.249655543	0.005678675
PYCARD	-1.202068741	0.030887698
GCLC	-1.181163339	0.029676262
ZNF662	-1.163813177	0.047971741
KITLG	-1.146198981	0.012651482
GSTA4	-1.141250126	0.024859655
STON2	-1.098860408	0.048847614
ARL3	-1.098185687	0.021176241
TUBA1A	-1.080380818	0.01169162
MTRNR2L11	-1.079106957	0.04121712
MCC	-1.053756421	0.014932427
SESN1	-1.041080423	0.004885901
PIK3R1	-1.032754895	0.030366764
TPRKB	-1.009871581	0.027551065

Legend: All genes with greater than 10-fold differential are listed. Genes with an estimated greater than 100-fold differential are illustrated in green. Genes that are associated with unadjusted p values <.01 or .05 are illustrated in red and blue, respectively. logFC: log fold change; P.Value: unadjusted P-values.

Supplementary figure 14: Associations of response with mIF cell density.

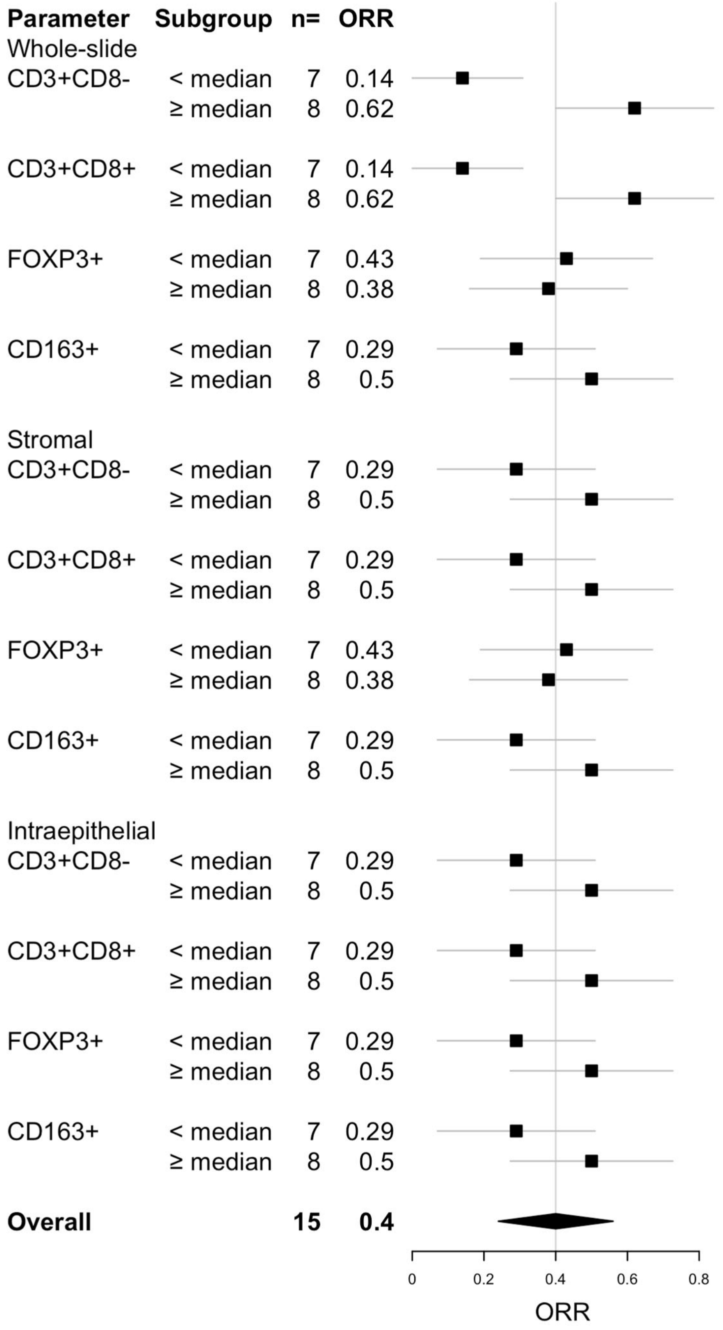

Legend: Intervals represent the 80% confidence interval of the point estimate of response rate. Statistical testing is not conducted given the exploratory nature of the assessment. ORR: overall response rate.

Supplementary figure 15: Baseline mIF stromal tumor infiltrates according to response

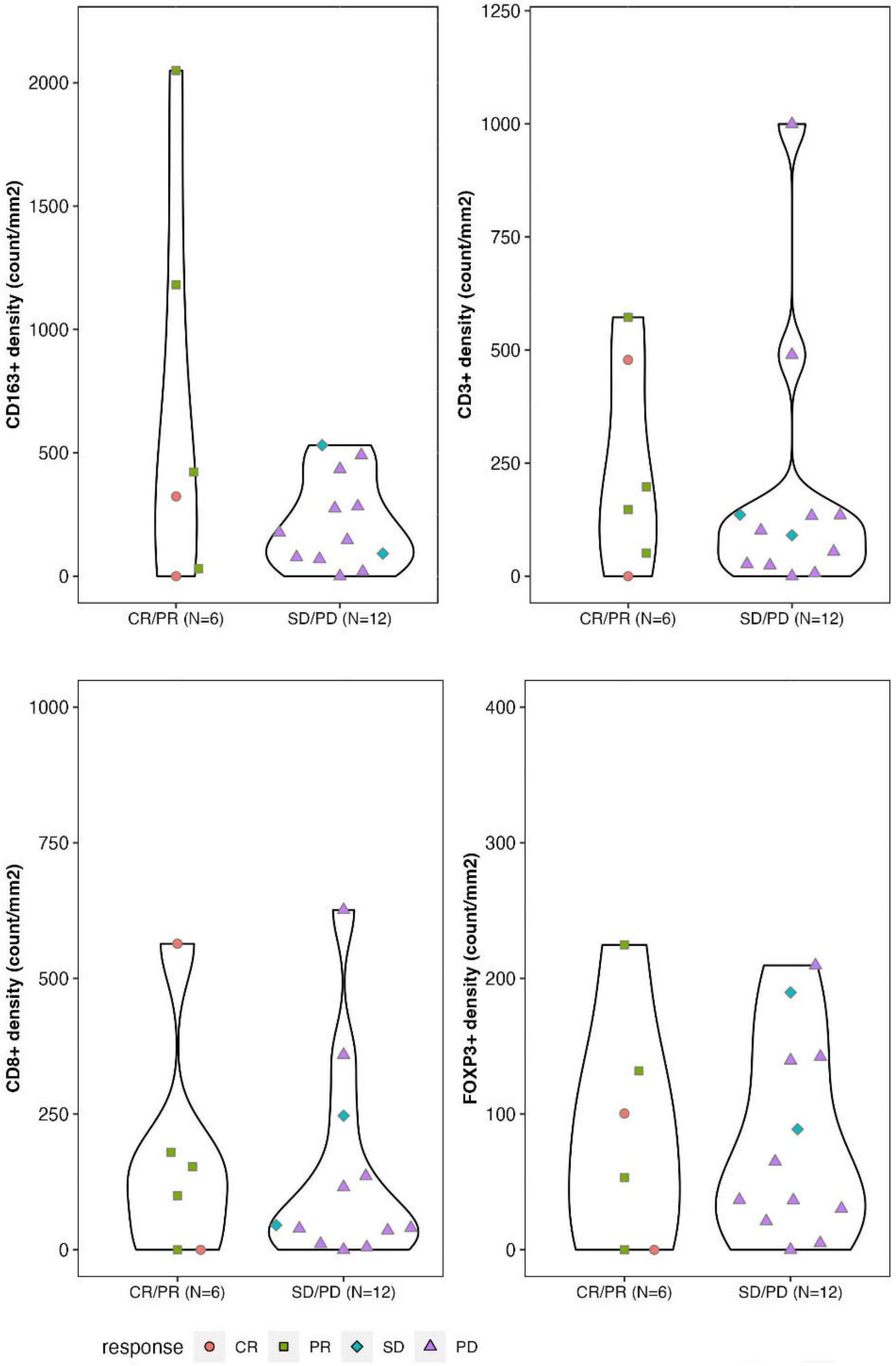

Legend: Distribution of baseline cell densities in the tumor stromal compartment are illustrated for patients with objective response versus not. CR: complete response; PR: partial response; SD: stable disease; PD: progression of disease.

Supplementary figure 16: Baseline mIF intraepithelial tumor infiltrates according to response

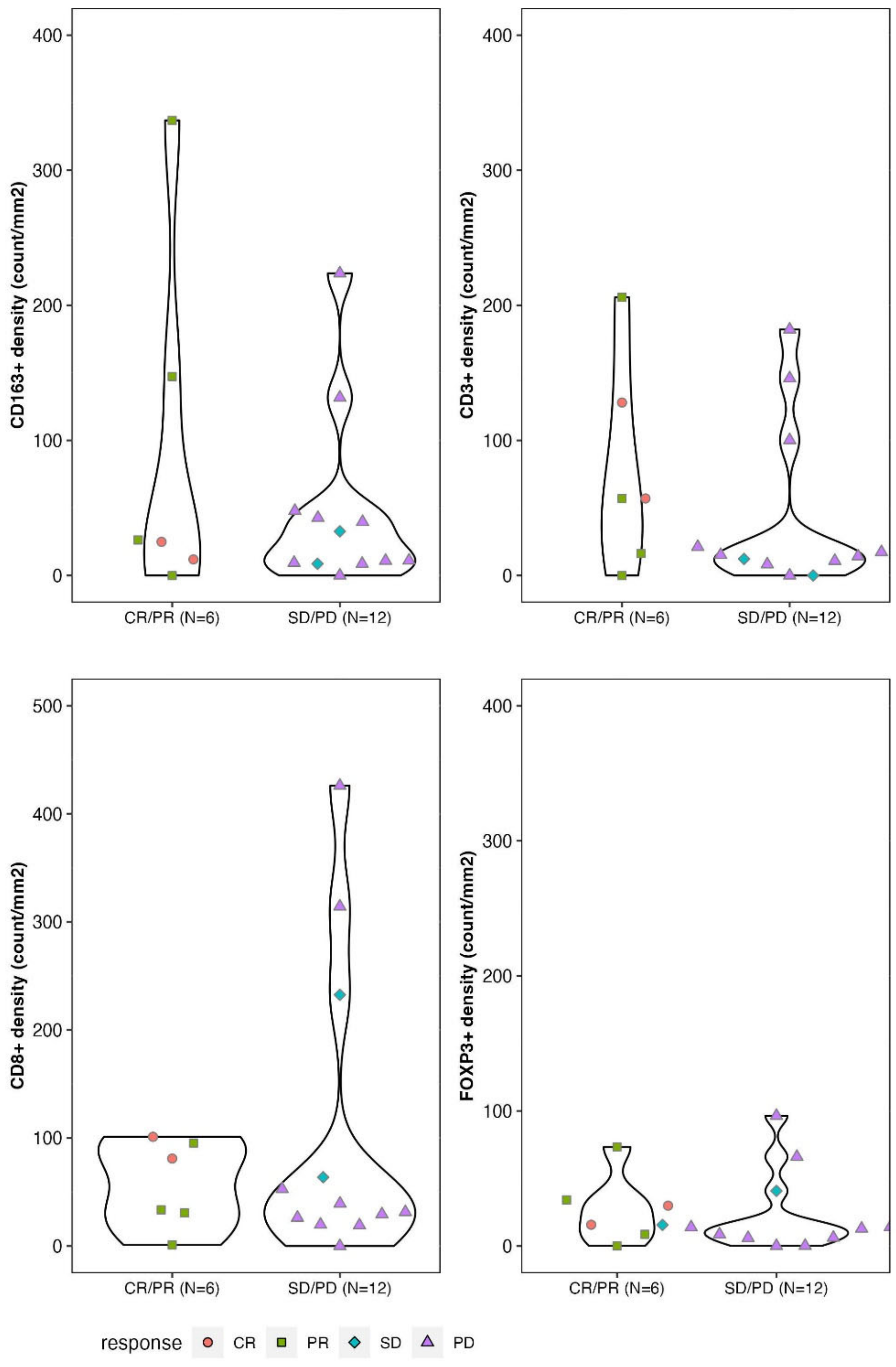

Legend: Distribution of baseline cell densities in the tumor intraepithelial compartment are illustrated for patients with objective response versus not. CR: complete response; PR: partial response; SD: stable disease; PD: progression of disease

Supplementary figure 17: Baseline mIF tumor infiltrates according to response (whole slide = stromal + intraepithelial).

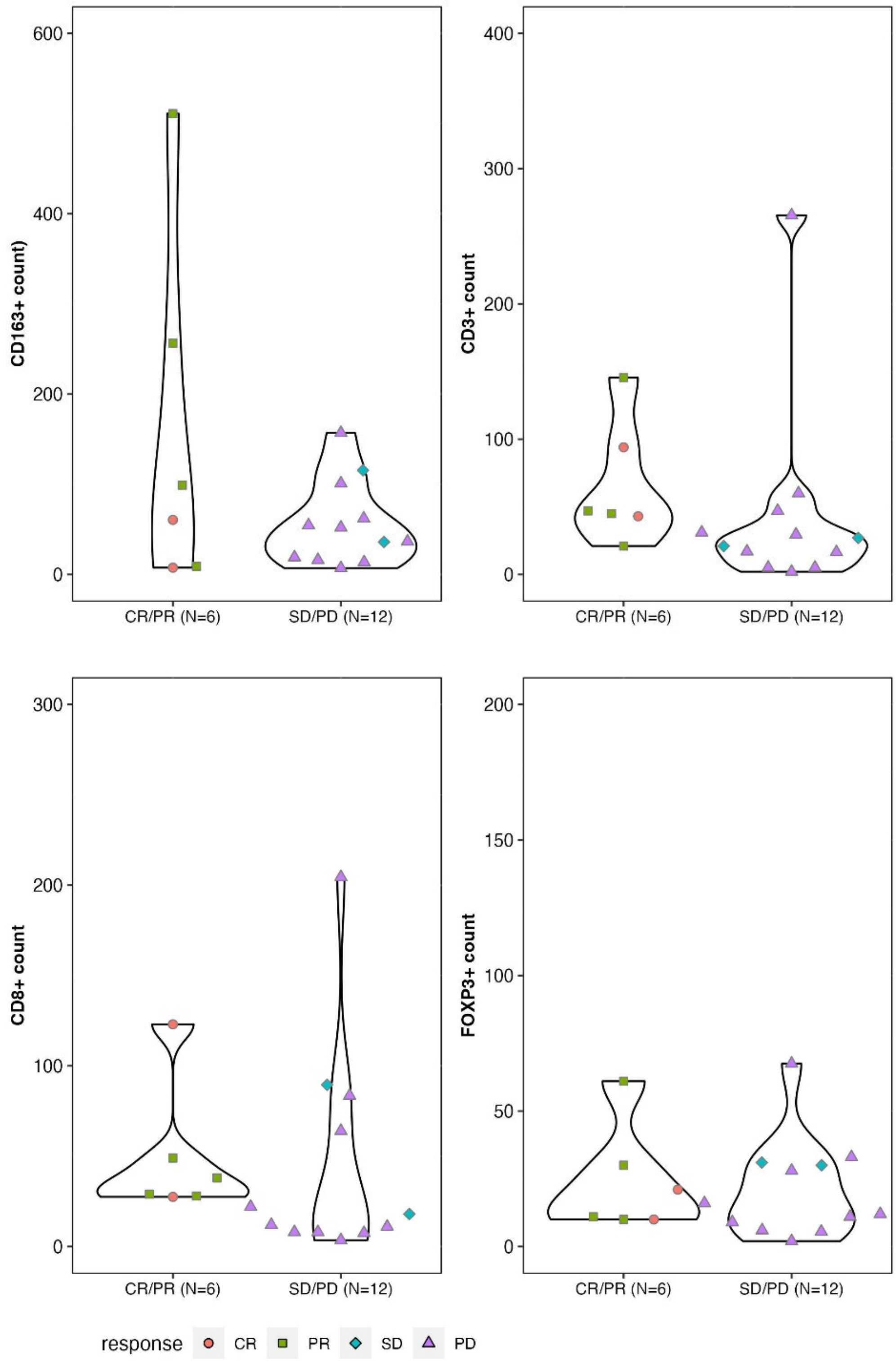

Legend: Distribution of baseline whole-slide cell counts are illustrated for patients with objective response versus not. There appears to be general trend of increased immune infiltrates amongst patients with objective response. CR: complete response; PR: partial response; SD: stable disease; PD: progression of disease.

Supplementary Figure 18: Effect of therapy on peripheral blood mononuclear cells

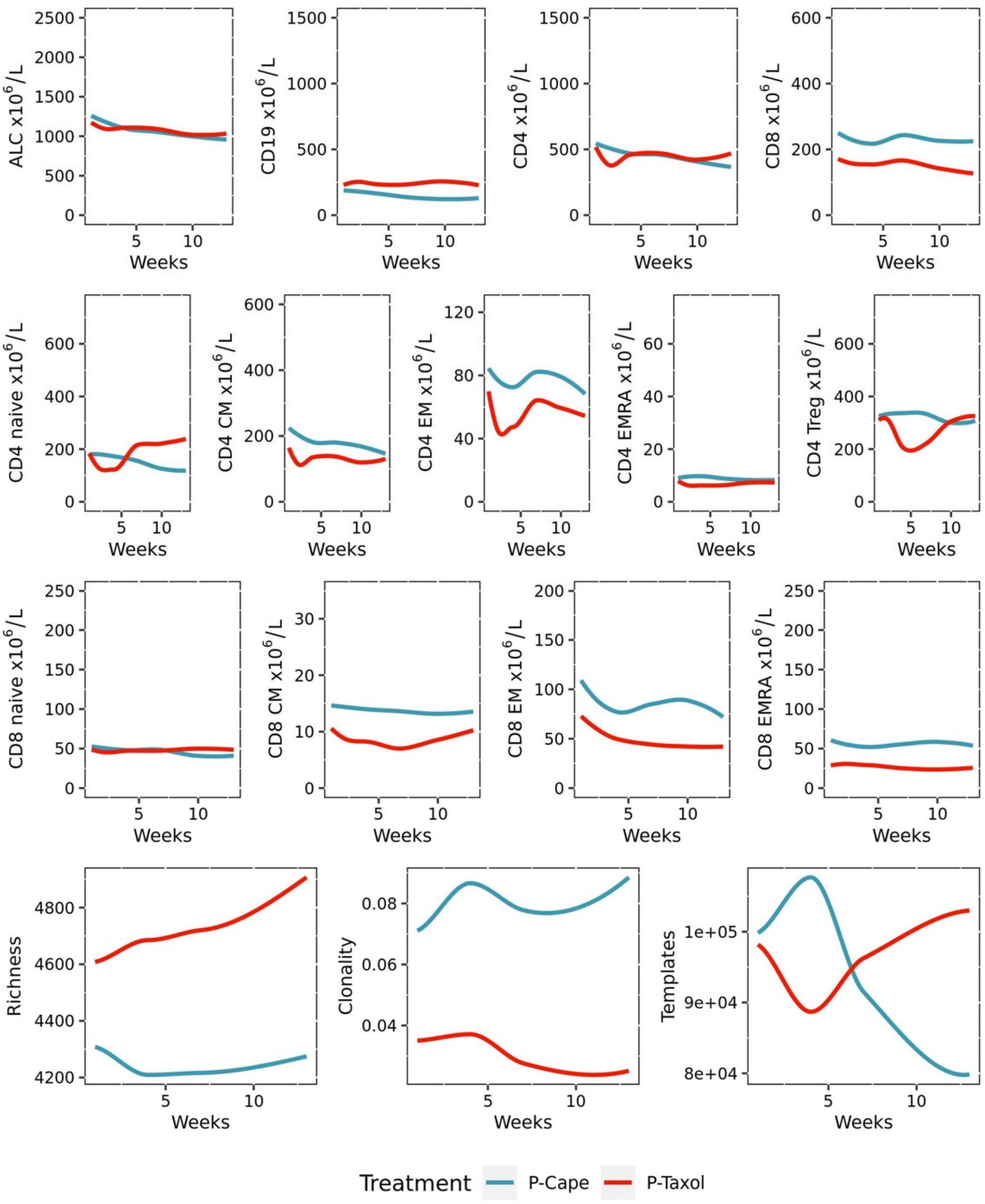

Legend: On-treatment LOWESS curves of peripheral lymphocyte counts as measured by routine clinical testing (ALC), multi-parametric real-time flow cytometry, and T-cell receptor DNA deep sequencing (TCR Richness, clonality, template copy number). ALC: absolute lymphocyte count; P-Cape: pembrolizumab + capecitabine; P-Taxol: pembrolizumab + paclitaxel; metastatic treatment with pembrolizumab plus capecitabine; Taxol: metastatic treatment with pembrolizumab plus weekly paclitaxel; TCR: T-cell receptor. Units: ALC: 10^3 cells/uL; Richness: # productive templates/ T-cell counts: cells/uL
