# Supplementary material for: A phase Ib trial of pembrolizumab plus paclitaxel or flat-dose capecitabine in 1st/2nd line metastatic triple-negative breast cancer
Source: NPJ Breast Cancer. 2023 Jun 21;9:53. doi: 10.1038/s41523-023-00541-2 (PMC10284878; doi:10.1038/s41523-023-00541-2)
Supplement: Supplementary file 2 — Reporting Summary [file 41523_2023_541_MOESM2_ESM.pdf]

nature portfolio

Corresponding author(s): Page DB

Last updated by author(s): Dec 15, 2022

Reporting Summary

Nature Portfolio wishes to improve the reproducibility of the work that we publish. This form provides structure for consistency and transparency in reporting. For further information on Nature Portfolio policies, see our Editorial Policies and the Editorial Policy Checklist.

Statistics

For all statistical analyses, confirm that the following items are present in the figure legend, table legend, main text, or Methods section.

n/a	Confirmed	
☐	☒	The exact sample size (*n*) for each experimental group/condition, given as a discrete number and unit of measurement
☐	☒	A statement on whether measurements were taken from distinct samples or whether the same sample was measured repeatedly
☐	☒	The statistical test(s) used AND whether they are one- or two-sided<br>*Only common tests should be described solely by name; describe more complex techniques in the Methods section.*
☐	☒	A description of all covariates tested
☐	☒	A description of any assumptions or corrections, such as tests of normality and adjustment for multiple comparisons
☐	☒	A full description of the statistical parameters including central tendency (e.g. means) or other basic estimates (e.g. regression coefficient) AND variation (e.g. standard deviation) or associated estimates of uncertainty (e.g. confidence intervals)
☒	☐	For null hypothesis testing, the test statistic (e.g. *F*, *t*, *r*) with confidence intervals, effect sizes, degrees of freedom and *P* value noted<br>*Give P values as exact values whenever suitable.*
☒	☐	For Bayesian analysis, information on the choice of priors and Markov chain Monte Carlo settings
☒	☐	For hierarchical and complex designs, identification of the appropriate level for tests and full reporting of outcomes
☐	☒	Estimates of effect sizes (e.g. Cohen's *d*, Pearson's *r*), indicating how they were calculated

Our web collection on statistics for biologists contains articles on many of the points above.

Software and code

Policy information about availability of computer code

Data collection	Microsoft word and R studio was used, but with no custom algorithms.
Data analysis	R studio was used for survival analysis, simple calculations, with no custom code. Linear modeling with R studio software package is mentioned in the methods.

For manuscripts utilizing custom algorithms or software that are central to the research but not yet described in published literature, software must be made available to editors and reviewers. We strongly encourage code deposition in a community repository (e.g. GitHub). See the Nature Portfolio guidelines for submitting code & software for further information.

Data

Policy information about availability of data

All manuscripts must include a data availability statement. This statement should provide the following information, where applicable:

- Accession codes, unique identifiers, or web links for publicly available datasets
- A description of any restrictions on data availability
- For clinical datasets or third party data, please ensure that the statement adheres to our policy

The clinical protocol and deidentified datasets generated during and/or analyzed during the current study are available from the corresponding author on reasonable request. RNA-seq data is available via the Gene Expression Omnibus (GEO) (accession number furnished upon acceptance of manuscript). TCR

sequencing data is available via Adaptive Biotechnologies repository (accession number furnished upon acceptance of manuscript). Raw flow cytometry data is available via flowrepository.org (accession number furnished upon acceptance of manuscript)

Human research participants

Policy information about studies involving human research participants and Sex and Gender in Research.

Reporting on sex and gender	One male was enrolled on study. This is mentioned in the manuscript. The low prevalance of males is due to the relative rarity of stage IV TNBC among the male population.
Population characteristics	This is reported in the demographics table.
Recruitment	As stated in the methods: "Subjects were recruited from local oncology clinics at both enrolling facilities. Emails were sent to regional clinic oncologists to ensure broad access to the clinical trials across various demographic groups."
Ethics oversight	As stated in the methods: "The trial was approved by the institutional review boards at Providence Cancer Institute (Portland, OR) and Cedars Sinai Medical Center (Los Angeles, CA), and was overseen by Providence Cancer Institute and the Earle A. Chiles Research Institute (Portland, OR). "

Note that full information on the approval of the study protocol must also be provided in the manuscript.

Field-specific reporting

Please select the one below that is the best fit for your research. If you are not sure, read the appropriate sections before making your selection.

☒ Life sciences ☐ Behavioural & social sciences ☐ Ecological, evolutionary & environmental sciences

For a reference copy of the document with all sections, see nature.com/documents/nr-reporting-summary-flat.pdf

Life sciences study design

All studies must disclose on these points even when the disclosure is negative.

Sample size	As stated in the methods: "This trial was powered to assess safety, defined as at least 60% of subjects in each arm completing two cycles of therapy without toxicity-related discontinuation of therapy or a dose delay exceeding ≥21 days. With a sample size of 14 patients per arm there was a 0.49 probability of declaring success assuming a true safety rate of 0.6, a 0.79 probability of declaring success assuming a true rate of 0.79, and a 0.96 probability of declaring success assuming a true rate of 0.9."
Data exclusions	In the manuscript details are provided as to why data from non-evaluable patients were excluded from efficacy analysis, but included in safety analysis when feasible. We also note that for biomarker studies some samples were excluded if they were not available (i.e. biopsy not feasible or viable).
Replication	Replication on clinical trials biopsy specimens is not possible because of scarcity of material and the risk of repeated biopsies posing harm to patients.
Randomization	The trial was not randomized.
Blinding	The trial was not blinded.

Reporting for specific materials, systems and methods

We require information from authors about some types of materials, experimental systems and methods used in many studies. Here, indicate whether each material, system or method listed is relevant to your study. If you are not sure if a list item applies to your research, read the appropriate section before selecting a response.

Materials & experimental systems

n/a	Involved in the study	
☒	☐	Antibodies
☒	☐	Eukaryotic cell lines
☒	☐	Palaeontology and archaeology
☒	☐	Animals and other organisms
☐	☒	Clinical data
☒	☐	Dual use research of concern

Methods

n/a	Involved in the study	
☒	☐	ChIP-seq
☐	☒	Flow cytometry
☒	☐	MRI-based neuroimaging

Clinical data

Policy information about clinical studies

All manuscripts should comply with the ICMJE guidelines for publication of clinical research and a completed CONSORT checklist must be included with all submissions.

Clinical trial registration	Trial registration: NCT02734290
Study protocol	The full protocol is offered upon request.
Data collection	These details are included in methods (locale) and in results (time of data collection).
Outcomes	Predefined outcomes are described in the text

Flow Cytometry

Plots

Confirm that:

☒ The axis labels state the marker and fluorochrome used (e.g. CD4-FITC).

☒ The axis scales are clearly visible. Include numbers along axes only for bottom left plot of group (a 'group' is an analysis of identical markers).

☒ All plots are contour plots with outliers or pseudocolor plots.

☒ A numerical value for number of cells or percentage (with statistics) is provided.

Methodology

Sample preparation	Peripheral blood mononuclear cells (PBMCs) were collected serially in heparinized tubes (subjects enrolled at EACRI) and Cytochex BCT tubes (all subjects). To avoid cellular loss and phenotypical changes due to cryopreservation, whole blood immune cells were analyzed either in real time on the day of collection (among subjects enrolled at EACRI) or within 24 hours of collection (among subjects enrolled at Cedars-Sinai). For TCR sequencing, DNA was prepared from sorted cell populations.
Instrument	BD LSRFortessa (BD Biosciences) was used for flow cytometric analysis of the samples.
Software	FACSDiva software was used to acquire flow cytometric data and FlowJo v10 was used for analysis.
Cell population abundance	N/A
Gating strategy	Live cells were gated on FSC/SSC (singlets), live/dead stain, and CD45+. CD3+ T cells and CD19+ B cells were then gated, followed by the indicated CD4+ and CD8+ T cell subsets, including naïve (CD45RA+/CCR7+), central memory (CM) (CD45RA-/CCR7+), effector memory (EM) (CD45RA-/CCR7-), effector memory cell re-expressing CD45RA (EMRA) (CD45RA+/CCR7-) T cells, and CD4+ T regulatory (CCR4+, CD127low, CD25high) cells (Tregs).

☒ Tick this box to confirm that a figure exemplifying the gating strategy is provided in the Supplementary Information.
